# Assessment of Allergenic Potential for Cross‐Reactivity of Olive ( *Olea europaea* ) Pit Xylo‐Oligosaccharide‐Rich Extract With Legislated Allergens in the European Union

**DOI:** 10.1002/fsn3.72207

**Published:** 2026-08-03

**Authors:** Maame Ekua Manful, Joseph O'Shea, Antía Torres, Paz Otero, Catherine Barry‐Ryan

**Affiliations:** ^1^ School of Food Science and Environmental Health Technological University Dublin Dublin Ireland; ^2^ Sustainability and Health Research Hub Technological University Dublin Dublin Ireland; ^3^ Food Safety Department Teagasc Food Research Centre Dublin Ireland; ^4^ Nutrition and Bromatology Group, Analytical Chemistry and Food Science Department, Faculty of Science Universidade de Vigo Ourense Spain

**Keywords:** allergenicity, ELISA, in silico, novel foods, olive pit, proteomics, xylo‐oligosaccharides

## Abstract

Novel food sources must undergo allergenicity assessments to ensure consumer safety and regulatory compliance. Xylo‐oligosaccharide‐rich extract (XOS) derived from the valorization of olive ( *Olea europaea* ) pit is categorized as a novel food in the context of European Union (EU) food laws. Despite the functional benefits of xylo‐oligosaccharides such as prebiotic, gut modulation, and weight management being a well‐established area, there is a lack of information on the allergenic potential of olive pit‐derived XOS extract. The study aimed to evaluate the cross‐reactivity of olive pit‐derived XOS extract with other allergens through both in vitro and in silico approaches. In vitro cross‐reactivity was assessed using enzyme‐linked immunosorbent assay (ELISA) against eight EU‐legislated allergens: wheat (gluten), milk, hazelnut, pistachio, soy, almond, lupin, and peanut. In silico allergenicity prediction was performed by analyzing the XOS extract amino acid sequence homology with known allergens. In the in vitro tests, hazelnut exhibited the highest cross‐reactivity (0–2.81 ppm), followed by gluten (0–2.10 ppm), almond (0–1.167 ppm), and pistachio (0.210–0.800 ppm). Milk showed minimal cross‐reactivity (0–0.07 ppm), while soy and lupin responses were below detection limits. The in silico analyses revealed that 81% of the 83 amino acid sequences showed no evidence of allergenicity, 7% showed weak evidence, and 12% indicated strong allergenic potential. The findings of the study suggest that olive pit‐derived XOS extract may contain proteins with the potential for cross‐reactivity with certain allergens, corroborating emerging concerns about fruit‐derived allergenic responses. Further clinical studies are recommended to generate additional insights on this preliminary investigation.

## Graphical abstract

Spray‐dried xylo‐oligosaccharide‐rich extract derived from olive pit endocarps was assessed for potential cross‐reactivity using in vitro ELISA and in silico approaches. The ELISA tests indicated potential cross‐reactivities with nuts (hazelnut, almond, pistachio, peanut), gluten, and milk in certain batches. The in silico analysis showed that 81% of the 83 amino acid sequences had no evidence of allergenicity, 7% showed weak evidence, and 12% indicated strong allergenic potential.

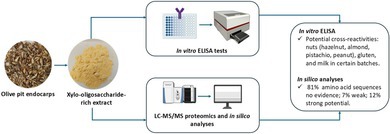

## 1 Introduction

The valorization of agricultural by‐products into functional food ingredients has gained considerable momentum in recent years, driven by sustainability and innovation in Agri‐food systems. Concurrently, the growing consumer demand for naturally derived functional food ingredients has contributed to the increased interest in alternative and novel food sources. Olive ( *Olea europaea* ) pits are a processing by‐product of olive mills with substantial valorization potential (Pantziaros et al. 2021). Traditionally, olive pits have been relegated to low‐value applications such as incineration for energy and fuel (Rodríguez et al. 2008). However, their lignocellulosic composition makes them a valuable feedstock for the production of sugars and matrices rich in xylo‐oligosaccharides (XOS) (Cordeiro et al. 2025; Mateo et al. 2021).

XOS are non‐digestible carbohydrates composed of xylose units joined by β‐1,4 glycosidic linkages (Ali et al. 2025). The functional properties of XOS have been well‐documented including prebiotic, weight management, gut modulation, antioxidant, anti‐tumoral, and anti‐inflammatory activities (Batsalova et al. 2022; Gufe et al. 2024; Li et al. 2021; Míguez et al. 2021). Despite their potential health benefits, the use of olive pit‐derived XOS extract as a novel food ingredient in the European Union (EU) requires comprehensive safety evaluation. According to EU Novel Food Regulation (EU) 2015/2283, any food not consumed to a significant degree before May 15, 1997, must undergo rigorous safety assessment prior to market authorization (European Commission 2015).

Although olives are not listed among the 14 major allergens legislated in the EU, reports of olive‐related allergic reactions have been documented. Olive allergy has been associated with olive pollen allergen (Ole e 1) and other allergenic proteins such as globulins, profilins, and thaumatin‐like proteins (Ole e 7) which have also been found to present in fruits such as peaches, cherries, and apples (Torres et al. 2017). According to the EFSA NDA Panel (EFSA Panel on Dietetic Products Nutrition and Allergies) (2014), fruit allergies are an ongoing concern in the EU and sensitized individuals may experience allergic reactions to these fruits due to potential cross‐reactivity with olive allergenic proteins (Barre et al. 2023; Palacín et al. 2012; Ünsel et al. 2009). Cross‐reactivity occurs when an immunoglobulin E (IgE) response to an allergenic molecule reacts with a different antigen due to shared structural (e.g., amino acid sequences, 3D structures), physicochemical properties, and physiological functions (Maurer‐Stroh et al. 2019).

Food allergenic responses could manifest as IgE‐mediated, non‐IgE mediated, and a combination of IgE and non‐IgE mediated occurrences (Connors et al. 2018). IgE‐mediated allergies are the most common type of food allergy with an acute onset, where antibodies (IgE) are triggered as a response mechanism (Zhang et al. 2021). The allergenic molecule covalently binds to IgE on mast cells and triggers the release of inflammatory biomarkers such as histamine. The IgE response manifests in various organs and systemic symptoms such as skin disorders (e.g., hives, oedema, and itching), respiratory disorders (wheezing), gastrointestinal tract disturbances (e.g., vomiting and diarrhea) and in extreme cases anaphylaxis. Non‐IgE‐mediated allergies are often sub‐acute or chronic responses characterized by a delayed onset with clinical symptoms primarily affecting the gastrointestinal tract and include protein‐induced enterocolitis, allergic proctocolitis, eosinophilic esophagitis, and celiac disease (Connors et al. 2018).

Assessing the allergenic properties of natural ingredients, especially non‐legislated products, is important due to the possible presence of immunogenic proteins (EFSA NDA Panel (EFSA Panel on Dietetic Products Nutrition and Allergies) 2014). Allergenicity assessments are commonly carried out through clinical trials and animal‐based studies. Other techniques are used for potential allergenicity analysis or for the detection of allergens and cross‐reactive molecules in food and feed. These include in vitro assays (such as ELISA and Western blotting), chromatographic techniques, mass spectrometry combined with sodium dodecyl sulphate polyacrylamide gel electrophoresis (SDS‐PAGE), and DNA‐based methods such as polymerase chain reaction (PCR) (Tuppo et al. 2022).

Clinical trials are often resource‐intensive (time, labor, and financial inputs) and are limited by the variability in individual allergenic responses. Animal models employed in pre‐clinical trials are associated with animal welfare concerns and policies have been implemented to phase out animal testing. Compared with clinical trials, in vitro and in silico systems are less expensive and can be used as screening tools to assess the potential allergenicity of molecules. While standard in vitro methods such as ELISA are relatively affordable, other commercial allergenicity kits (e.g., cell‐based functional assays) can be costly, and only a limited number of validated commercial kits are available.

The ELISA technique is one of the commonly employed in vitro allergenicity screening tools (Polonschii et al. 2024). In principle, the allergenic molecule binds to an enzyme‐coupled antibody, and in the presence of a specific substrate enzyme, a colorful and measurable product is formed. Direct, indirect, and sandwich ELISA techniques are employed. Both the direct and indirect ELISA share the commonality of an immobilized antigen, followed by an enzyme‐conjugated antibody (in the direct assay) and a primary and secondary antibody (with enzyme‐linked secondary antibody) in the indirect assay. The sandwich ELISA, which is the most common technique, differs by the immobilization of the primary antibody. In the technique, the allergenic molecule (antigen) is “sandwiched” between the capture immobilized antibody and the enzyme‐linked antibody. In this context, ELISA can be used to verify cross‐reaction of particular molecules with target allergens.

In silico predictions have emerged as a complimentary rapid tool for the theoretical assessment of allergenicity of proteins based on their proteomic profiles. Various computational methods for predicting protein allergenicity including AllergenOnline, AllerHunter, Structural Database of Allergenic Proteins (SDAP), AllerTOP, and BIOPEP have been reported (Hayes et al. 2015). AllerCatPro 2.0 is a bioinformatic tool developed in 2019 by researchers from the Bioinformatics Institute at the Agency for Science, Technology, and Research in Singapore for protein allergy prediction based on their biophysical properties (Maurer‐Stroh et al. 2019). The AllerCatPro web server (https://allercatpro.bii.a‐star.edu.sg/) predicts protein allergenicity using their amino acid sequences and 3D protein structure by comparison with a dataset of over 5000 known allergens sourced from databases originating from WHO/International Union of Immunological Societies, UniProtKB, Allergome, Food Allergy Research and Resource Program and Comprehensive Protein Allergen Resource (Nguyen et al. 2022). In silico models are important for prioritizing molecules that need further testing (e.g., amino acid sequence homology identification above 35% in 80 amino acid sequences).

Allergenicity predictions are often determined based on protein molecular structure and associated biological properties in preliminary instances. According to the EFSA NDA Panel, such predictions are not sufficient. They must be accompanied by in vitro immunological and clinical assessments. This study aimed to assess the allergenic potential of valorized olive‐pit xylo‐oligosaccharides using in vitro immunological assays (ELISA) and in silico prediction tools (AllerCatPro), based on their amino acid sequences.

## 2 Materials and Methods

### 2.1 Samples

Three independently produced xylo‐oligosaccharide‐rich (XOS) samples (Batches 1, 2, and 3) were obtained from Isanatur (Navarra, Spain) for analyses. XOS is a complex mixture with commercial purity ranging from 75% to 95% (Aachary and Prapulla 2011). The samples were derived from olive pits separated from seeds, taken through autohydrolysis, followed by concentration and spray‐drying to obtain xylo‐oligosaccharides‐rich powder with 0.55% to 0.64% protein content (according to the Dumas technique).

### 2.2 In Vitro Allergenicity Determination Through the Enzyme‐Linked Immunosorbent Assay (ELISA)

Eight commercial sandwich ELISA allergenicity test kits were purchased from Romer Labs GmbH (Getzersdorf, Austria). The ELISA kits consisted of AgraQuant Almond, Peanut, Hazelnut, Lupin, Soy, Gluten, Pistachio, and Milk. In the absence of a commercial positive control, ingredients (almonds, peanuts, lupin seeds, soy milk, pistachio, wheat flour, and dairy milk) known to contain the allergenic proteins were purchased from the Irish Market (Tesco, Dublin, Ireland) and used as laboratory‐prepared positive controls.

#### 2.2.1 Sample Extraction

Sample extraction was performed according to the manufacturer's instructions with slight variations depending on the ELISA kit. For all kits except milk and gluten, 1 g of homogenized sample was added to 20 mL extraction buffer, vortexed and kept in an OLS 200 shaking water bath (Grant, Royston, United Kingdom) at 60°C for 15 min. For the milk kit, 0.5 g of sample was suspended in 10 mL of extraction buffer and shaken for 15 min at room temperature. For the gluten kit, 1 g of sample was suspended in 10 mL of extraction buffer and shaken for 5 min at room temperature. Following extraction, all suspensions were centrifuged at 2000 *× g* for 10 min, and the supernatants were filtered through a 0.45 μm filter prior to analysis.

#### 2.2.2 Enzyme‐Linked Immunosorbent (ELISA) Allergenicity Assay

A 100 μL sample extract was added to antibody‐coated microwells and incubated at room temperature for 20 min to allow reaction with the allergen in samples. The wells were emptied and washed with 100 μL wash buffer five times to remove any residue. Enzyme‐antibody conjugate solution (100 μL) was added to each well and incubated for 20 min to allow the antigen–antibody reaction. The wells were emptied and washed five times. The enzyme substrate solution (100 μL) was added to each well and incubated for 20 min. An acidic solution was added to each well to terminate the reaction with a visible color change from blue to yellow. The absorbance of the solutions was read at 430 nm (with a reference wavelength of 630 nm) with a microplate reader (BioTek Instruments, Vermont, USA). Experiments were performed in duplicate. The same procedure was repeated for calibration standards and the respective positive controls. The allergenic protein concentrations (ppm) in the samples were extrapolated from the prepared respective calibration curves.

### 2.3 Peptide Sequence Identification in Olive Pit Xylo‐Oligosaccharides

#### 2.3.1 Sample Preparation and Protein Digestion

Sample (1 g) was homogenized in 25 mL methanol: chloroform solution (1:2) and vortexed for 1 min. The mixture was sonicated (Argo Lab DU‐65, Carpi, Italy) for 10 min to enhance lipid separation and was centrifuged (Kubota 6900, Tokyo, Japan) for 5 min at 6260 × *g*. The supernatant was discarded, and 100 mg of the pellet was resuspended in 1 mL of Tris–HCl (0.5 M, pH 6.8), 10% sodium dodecyl sulphate (SDS), and 0.3% dithiothreitol (DTT) (1:1:1).

#### 2.3.2 Protein Precipitation

100 μL of trichloroacetic acid (TCA, 100%) was added to the suspension, vortexed, and left on ice for 30 min to allow protein precipitation. The mixture was centrifuged at 10,000 × *g* for 5 min and the supernatant was carefully aspirated. The resulting pellet was washed several times with 500 μL cold acetone (4°C) until the solution was clear after centrifugation at 10,000 × *g* (at 4°C) between washes.

##### 2.3.2.1 Gel Electrophoresis and In‐Gel Digestion

The washed pellet was resuspended in 20 μL of Tris–HCl (0.5 M, pH 6.8) and 10% SDS and loaded onto a standard Laemmli‐type polyacrylamide gel allowing the proteins to stack and enter the resolving gel, but not to separate. The gel bands were excised and washed with 25 mM ammonium bicarbonate, then with 50% acetonitrile (ACN)/25 mM ammonium bicarbonate in an ultrasonic bath. The proteins were then reduced with 10 mM DTT for 1 h and alkylated with 55 mM iodoacetamide for 30 min. The digestion was carried out by incubating with 40 ng of trypsin (Promega V5280) at 37°C overnight, and tryptic peptides were extracted from the gel matrix in two steps with 0.5% trifluoroacetic acid and 100% ACN.

#### 2.3.3 Peptide Identification and Database Search

The tryptic peptides were dried in a speed‐vacuum concentrator at 45°C (Concentrator plus, Eppendorf, Hamburg, Germany), reconstituted in LC/MS‐grade water containing 0.1% (*v*/*v*) formic acid, and analyzed by electrospray ionization tandem mass spectrometry on a hybrid high‐resolution LTQ‐Orbitrap Elite mass spectrometer coupled to an Easy‐nLC 1000 UHPLC system (Thermo Fisher Scientific, Waltham, MA, USA).

The peptides were transferred to a reverse phase column (PepMap RSLC C18, 2 μm, 100 Å, 75 μm × 50 cm, Thermo Fisher Scientific Waltham, MA, USA) and eluted with an acetonitrile gradient of 5%–35% containing 0.1% formic acid for 120 min at a flow rate of 300 nL/min. The resulting elutes were directly submitted into the mass spectrometer, which was set in positive ion mode in a data‐dependent mode. A full MS scan was performed from 390 to 1700 m/z and a resolution of 120,000. A tandem mass spectrometry (MS/MS) scan was then performed with the top 15 most intense ions at 35% of the normalized collision energy (NCE), with a dynamic exclusion time of 30 s, a minimum signal threshold of 1000.0 and an isolation width of 2.0 Da.

The MS/MS spectra were processed using Thermo Scientific's Proteome Discoverer which scanned the peptide spectra against peptide sequences in the UniProtKB (https://uniprot.org/) database to identify the parent allergenic proteins and their amino acid sequences. The database search was restricted to the *Olea europaea* taxonomy (taxon ID: 4145) due to the source material (olive pits). As of 5th June 2024, the UniProtKB database contained 81,312 deposited protein sequences (of which 110 are reviewed SwissProt entries) related to the queried taxon (ID: 4145). All three XOS batches (1, 2, and 3) were subjected to peptide sequencing following the previously described protocol. The identified peptides were mapped to their corresponding (parent) proteins, and their accession numbers were retrieved for subsequent analyses. Batch 2 yielded the highest number of identified proteins (*n* = 83 sequences) and included similar proteins detected in batches 1 and 3. Batch 2 was therefore selected for the in silico allergenicity prediction. The complete datasets for all three batches are provided in Data S1 (Tables S1–S3).

### 2.4 In Silico Allergenicity Prediction With AllerCatPro 2.0

The protein accession numbers from batch 2 (*n* = 83 sequences) were used to retrieve the complete protein (amino acid) sequences from UniProtKB in FASTA format. The FASTA text of each protein sequence was submitted as queries to the AllerCatPro server (allercatpro.bii.a‐star.edu.sg). Similarity data among the queried amino acid sequences with the linear sequence and 3‐dimensional epitopes of known allergens, gluten‐like Q repeats, autoimmune allergens, low allergenic proteins, and similarity with 3 × 6‐mers were analyzed.

## 3 Results and Discussion

### 3.1 In Vitro Cross‐Reactivity Potential of Olive Pit Xylo‐Oligosaccharides

ELISA testing is a commonly employed in vitro technique due to its simplicity, sensitivity, and readouts in a shorter time. The technique can detect trace amounts of allergens in food products and is useful in ensuring food safety and compliance with allergen labelling regulations. Additionally, it can serve the purpose of identifying cross‐reactivity of conventionally non‐allergenic matrices to known allergens.

The eight EU‐legislated allergen ELISA antigens analyzed showed varied cross‐reactivities with the olive pit XOS extract (Table 1). The highest cross‐reactivity was found with hazelnuts (0–2.81 ppm), followed by gluten (0–2.10 ppm) and other nuts: almond (0–1.17 ppm) and pistachio (0.210–0.80 ppm). Additionally, the lowest cross‐reactivity was exhibited with milk, while soy and lupin responses were below the detection limits. Similarly, the peanut cross‐reactivity was below the detection limit, except in a single batch.

**TABLE 1:** In vitro ELISA allergenic responses of olive pit xylo‐oligosaccharides extract (XOS).

Ingredient	Batch	Allergenic response (ppm)							
		Peanuts	Gluten	Soy	Pistachio	Almond	Hazelnut	Milk	Lupins
XOS	1	a	a	a	0.21	0.25	1.81	0.33	a
	2	1.44	a	a	0.80	a	a	a	a
	3	> 40	2.10	a	0.25	1.17	2.81	0.07	a
Positive controls		> 40	> 120	> 1000	> 40	> 10	> 40	> 10	> 30

^a^ Below detection limits. Positive controls: Store ingredients for test kits (almonds, peanuts, lupin seeds, soy milk, pistachio, wheat flour, and dairy milk).

Olive fruits are not known to be major allergens. Despite its wide consumption, few reports have documented its in vitro and in vivo allergenicity potential. Olive fruit allergenicity has been mostly associated with olive pollen allergens (e.g., Ole e 7 and Ole e 10). Olive pollen allergy is an IgE‐mediated response affecting about 20% of the Mediterranean population (Barral et al. 2005). The allergy manifests as a respiratory allergic reaction and is common among olive mill factory workers (Palomares et al. 2008; Torres et al. 2014). Individuals allergic and sensitized to olive proteins are likely to experience cross‐reactivity to nuts due to the lipid transfer protein syndrome (Ünsel et al. 2009; Vicente et al. 2021). Lipid transfer proteins are allergenic proteins present in fruits, vegetables, and nuts which are associated with plant development and stress response (Barre et al. 2023).

Over the past decades, allergenic proteins such as oleosins and thaumatin‐like proteins have been identified in olives (Palomares et al. 2008). Oleosins are plant oil bodies and have been reported to be present in olives, almonds, and other nuts and fruits belonging to the *Rosaceae* family (Alvarez‐Eire et al. 2012; Montealegre et al. 2014). Additionally, thaumatin‐like proteins have been found in wheat (Palacín et al. 2012). These findings indicate a potential cross‐reactivity between the olive‐derived ingredients and allergenic proteins found in nuts and wheat.

While these findings are insightful, several factors can play a role in allergenicity, especially individual variable responses. According to the EFSA NDA Panel (EFSA Panel on Dietetic Products Nutrition and Allergies) (2014), not all cross‐reactions identified in in vitro assays have clinical significance. With the advent of increasing fruit allergy over the years, employing in vitro testing to identify potential cross‐reactivity and lipid transfer protein allergic reactions is important for consumer health protection.

### 3.2 Amino Acid Sequence Characteristics of Olive Pit Xylo‐Oligosaccharides

The amino acid sequencing analysis of the peptide fractions associated with the XOS extract derived from olive stones revealed a broad, heterogeneous, and functionally diverse proteome across all batches studied. The number of sequences varied across batches, with 39, 83, and 30 sequences identified in batches 1, 2, and 3, respectively (Data S1, Tables S1–S3). However, 15 protein sequences were consistent across all three batches (Data S1, Tables S1–S3). This batch‐to‐batch variation is typical for independently produced plant‐derived XOS extracts due to matrix complexity and seasonal differences of the material of origin (olive pits).

Numerous regulatory proteins of nuclear origin were identified, including histones, transcription factors, and zinc‐finger domain proteins, along with structural cytoskeletal components such as actins and myosins. Likewise, a wide variety of metabolic enzymes associated with energy pathways, redox processes, and biosynthetic routes were detected, as well as proteins related to stress response, indicating that the XOS extract contained a wide range of functional proteins originating from the olive pit. Finally, a notable proportion of the sequences corresponded to proteins that have not yet been characterized in *Olea europaea* , reflecting the still incomplete state of its functional genome annotation. A representative selection of the proteomic profile of XOS extract is presented in Table 2 to illustrate the functional diversity of the identified proteins, with the complete dataset of all 83 sequences provided in the Supporting Information.

**TABLE 2:** Selection of identified proteins in olive pit xylo‐oligosaccharide samples^a^.

Accession	Description	Coverage [%]	# AAs	Sequence	Molecular function
A0A8S0PCB3	Elongation factor 1‐alpha [OS = *Olea europaea* subsp. europaea]	2	483	IGGIGTVPVGR	Nucleic acid binding activity; GTPase activity; Translation elongation factor activity
A0A8S0PMV2	Submitted name: Uncharacterized protein [OS = *Olea europaea* subsp. europaea]	2	504	STTAVLDLLPR	—
A0A8S0PS52	Submitted name: Actin‐7 [OS = *Olea europaea* subsp. europaea]	7	377	AGFAGDDAPR; VAPEEHPVLLTEAPLNPK	ATP binding; Hydrolase activity
A0A8S0QBD8	Proteasome subunit alpha type [OS = *Olea europaea* subsp. europaea]	6	249	LTVEDPVTVEYITR	—
A0A8S0QCC2	Submitted name: GTPase Era [OS = *Olea europaea* subsp. europaea]	2	437	KLEWYEK	Nucleic acid binding activity
A0A8S0QIX0	Submitted name: Transcription factor bHLH18‐like [OS = *Olea europaea* subsp. europaea]	3	348	FSDKDVLIR	Protein dimerization activity
A0A8S0R4B0	Fructose‐bisphosphate aldolase [OS = *Olea europaea* subsp. europaea]	2	689	GILAADESTGTIGK	Fructose‐bisphosphate aldolase activity
A0A8S0TEC5	Submitted name: Heat shock cognate 70 kDa 2‐like [OS = *Olea europaea* subsp. europaea]	2	679	IINEPTAAAIAYGLDK	ATP binding; ATP‐dependent protein folding chaperone
A0A8S0SJ95	Histone H2A [OS = *Olea europaea* subsp. europaea]	11	151	HLQLAIR; AGLQFPVGR	Nucleic acid binding activity; Protein heterodimerization activity; Structural constituent of chromatin
A0A8S0UEF5	Structural maintenance of chromosomes protein [OS = *Olea europaea* subsp. europaea]	1	1247	NEAEAYMLK	ATP binding; ATP hydrolysis activity
A0A8S0UQW6	Submitted name: Actin [OS = *Olea europaea* subsp. europaea]	5	377	AGFAGDDAPR; GYSFTTTAER	ATP binding; Hydrolase activity

^a^ Full proteomic profile is available in the Supporting Information.

These findings provide valuable information about the structural and functional properties of the proteins associated with olive pit XOS extract. The analysis of these sequences can reveal conserved motifs or structural domains that may influence stability, enzymatic activity, or interactions with other biomolecules. In addition to the allergenic potential studies, characterizing these amino acid patterns is essential for understanding the biochemical behavior of the proteome associated with the XOS extract, predicting potential bioactivities, and guiding future functional applications.

No studies analyzing specifically the proteome of XOS derived from olive stones have been found, although other olive by‐products have been investigated. For example, Esteve et al. (2012) analyzed the proteome of olive seeds and pulp, detecting storage proteins, oleosins, and a high number of histones in the seed, while identifying defense‐related proteins, fatty acid biosynthesis enzymes, and carbon‐metabolism proteins in the pulp. Compared with these results, the protein profile of the XOS extract from olive stones is more diverse and heterogeneous, possibly because it combines characteristics from both tissues.

The presence of chaperones and antioxidant enzymes, such as catalase, indicates that the samples in this study were subjected to mechanical or oxidative stress during extraction, triggering protective mechanisms, as reported in previous studies (Araújo et al. 2021; Kazemi Oskuei et al. 2025). This pattern is reflected in the XOS extracts' protein profile, suggesting that this protein matrix may preserve evidence of adaptive responses to stress during processing. Although information about this specific protein matrix is limited, several studies have shown that the activation of chaperones and antioxidant systems constitutes a protective mechanism against stress in other plant species, such as wheat (Ravindra et al. 2025) and tomato (Xu et al. 2024).

A higher proportion of the proteins identified in the *Olea europaea* proteome have not yet been characterized, representing an opportunity for future research on their biological relevance and potential biotechnological applications. Additional analyses are also needed to assess how the peptide profile may influence the stability, bioactivity, or functional properties of XOS. Taken together, these results provide a solid foundation for exploring future functional applications of XOS and for conducting comparative studies with other plant‐based by‐products.

### 3.3 In Silico Allergenicity Prediction of the Amino Acid Sequences in Olive Pit Xylo‐Oligosaccharides

Allergen prediction is important for the early screening of potential allergens before clinical or in vivo trials. According to the World Health Organization (WHO), a sequence homology screening is important for early allergenicity prediction prior to the conduct of further tests (World Health Organization et al. 2001). Computational tools are employed in identifying similarities between the primary, secondary, and tertiary protein structures of novel proteins and known allergens.

Olive pit XOS extract is a novel food according to the EU regulatory definition with limited information on its allergenicity. The positive allergenic responses in the in vitro ELISA tests in one or two XOS extract batches suggest a potential cross‐reactivity with the relevant known allergens (Table 1). The literature is limited in the allergenic potential of xylo‐oligosaccharides derived from other sources (wheat, birchwood, etc.). As a novel food, it is essential to characterize its potential allergenic properties.

The AllerCatPro allergenicity prediction categorizes outputs into three profiles: strong evidence for allergenicity, weak evidence for allergenicity, and no evidence for allergenicity. Strong evidence for allergenicity is predicted when the percent similarity between the 3‐D epitopes of the query amino acid sequence and those of known allergens is greater than 93%. Additionally, the percent similarities within a window of 80 amino acid sequences above 35% predict strong evidence for allergenicity (World Health Organization et al. 2001). A third criterion evaluates the similarity between every three repetitive short amino sequences (3 × 6‐mers). When this third criterion is not met, weak evidence for allergenicity is predicted (Nguyen et al. 2022).

The XOS extract was predicted to possess a low allergenic profile. About 19% (16 out of 83) of the amino acid sequences exhibited similarities with known allergens from other species, presented in Table 3 (Data S2 and S3). These predictions ranged from strong to weak evidence for allergenicity. The allergenic protein hits were from fish (iridescent shark), cereals: wheat and rice, fungal species: *Aspergillus* and *Penicillin* spp., rubber tree or latex, ragweed, and mite (house mite, mold mite, and groceries or sugar mite).

**TABLE 3:** Similarity of olive pit xylo‐oligosaccharides amino acid sequences to known allergens across species.

Allergen	Species name	Species common name	No of hits	Similarity in linear 80 AA sequence (%)	Similarity with 3D epitope (%)	Prediction
Tyr p 28	*Tyrophagus putrescentiae*	Mold mite	15	91.2	100	Strong evidence
Tyr p 28	*Tyrophagus putrescentiae*	Mold mite	15	92.5	100	Strong evidence
Amb a 12	*Ambrosia artemisiifolia*	Ragweed	37	97.5	100	Strong evidence
Hev b HSP80	*Hevea brasiliensis*	Rubber tree	3	98.8	100	Strong evidence
Hev b HSP80	*Hevea brasiliensis*	Rubber tree	3	65	81.2	Weak evidence
Hev b HSP80	*Hevea brasiliensis*	Rubber tree	3	100	100	Strong evidence
Katanin	*Triticum aestivum*	Wheat	3	53.8	—	Strong evidence
Tri a 23kd	*Triticum aestivum*	Wheat	1	38	—	Strong evidence
Der f 32	*Dermatophagoides farinae*	House dust mite	2	38.8	—	Strong evidence
Der f EF	*Dermatophagoides farinae*	House dust mite	1	78.8	—	Strong evidence
Pan h 3	*Pangasianodon hypophthalmus*	Iridescent shark	9	77.5	93.33	Strong evidence
Lep d 33	*Lepidoglyphus destructor*	Groceries mite, sugar mite	6	55	76.9	Weak evidence
ATP synthase subunit beta, mitochondrial	*Penicillium glabrum*	*Penicillium* spp.	1	91.5	—	Weak evidence
Asp f RPS3	*Aspergillus fumigatus*	*Aspergillus* spp.	2	86.2	92.9	Weak evidence
Cla h 10	*Cladosporium herbarum*	*Cladosporium* spp.	6	50	61.5	Weak evidence
Unknown	*Oryza sativa*	Rice	3	46.2	64.7	Weak evidence

The amino acid sequence homology of the XOS extract with allergens such as katanin and Tri a 23kd (from *Triticum aestivum* or wheat) and Pan h 3 (from *Pangasianodon hypophthalmus*; iridescent shark fish) showed strong evidence of allergenicity. These sequences have a high similarity with the XOS queried amino acid sequences in both their linear structures (above 90%) and in the 3D epitopes (100%). Fish and wheat belong to the list of EU‐regulated allergens due to their well‐established allergenicity profile (European Commission 2006). The predicted shared similarities between the linear sequences and 3D epitopes of XOS extract proteins with wheat allergens are consistent with the results obtained in the in vitro ELISA test, which showed a positive response for wheat allergens (Table 1). Additionally, strong evidence of allergenic cross‐reactivities between the XOS amino acid sequences with ragweed and rubber tree allergens was predicted. Latex, olive pollen, and ragweed pollen contain proteins that belong to the profilin family, suggesting a potential cross‐reactivity (Gromek et al. 2024; Nguyen et al. 2022).

Five out of the 16 allergenic amino acid sequences were estimated to have weak evidence for allergenicity due to the similarities in 3D structures being less than 93%. These included allergens found in grocery mites (Lep d 33), *Penicillin* spp. (ATP synthase), and *Cladosporium* spp. (Cla h10). Alvarez‐Eire et al. (2012) reported on olive fruit‐induced anaphylaxis in a patient with historic house dust mite allergy. Additionally, there is limited data in the literature about olive allergens and their cross‐reactivity with fungal allergenic proteins. In this case, environmental manufacturing conditions could play a role in exposing XOS to fungal allergens.

There was no similarity with gluten‐like proteins in the XOS sample amino acid sequences. Additionally, there were no 3 × 6‐mer overlapping short sequences associated with known allergens. The in silico allergenicity predictions suggest a potential cross‐reactivity of olive to latex, ragweed, wheat, fish, and fungal allergenic proteins. These findings further suggest potential risks of cross‐reactivity in individuals allergic to proteins homologous to the XOS extract's amino acid sequences when present in doses sufficient to trigger an allergic reaction. A low allergenic profile was found for the XOS extract since 81% of the amino acid sequences present showed weak evidence for allergenicity.

These insights are relevant for providing information to consumers in ensuring consumer protection. While ongoing research and policies have attempted to establish safety and regulatory thresholds for these allergens, there is limited regulatory information on the recommended thresholds of cross‐reactive (non‐native) proteins. For example, the EU established a gluten limit of 20 ppm in products that are to be marketed as “gluten‐free” (European Commission 2014). In their human feed studies, Catassi et al. (2007) recommended a maximum dose of 50 g/L gluten for celiac patients. The Joint FAO/WHO Food Standards Program has recently established reference doses of common allergens (Food and Agriculture Organization of the United Nations (FAO) 2024). These include 1 mg protein (total protein from the allergen) for almond and pistachio, 2 mg for peanut and milk, 3 mg for hazelnut, and 10 mg for soy and lupin. It remains unclear if these values are limited to the native proteins or cross‐reactive proteins.

## 4 Conclusion

The combined in vitro immunological, proteomics, and in silico studies revealed low allergenic potential for olive pit xylo‐oligosaccharides extract. A potential in vitro cross‐reactivity was observed with allergenic proteins in gluten (wheat), milk, and nuts (almond, hazelnut, pistachio, and peanut). Furthermore, the in silico analyses revealed sequence homology with known allergens (wheat, mites, and fish) with an overall low allergenicity risk. In vitro assays and in silico tools are complementary approaches and can provide early‐stage allergenicity screening to support decision‐making and prioritize samples prior to clinical testing. However, not all in vitro allergenic reactions have clinical significance. In silico predictions depend on existing allergen databases which can lead to false negatives for novel proteins. Clinical studies are recommended to further support in vitro and in silico findings. In addition, de novo protein sequencing for unknown or novel proteins is critical to fill database gaps. Batch‐to‐batch variability observed is an important consideration in non‐clinical allergenicity testing. While the valorized olive pit XOS extract was found to possess minimal allergenic potential, further clinical testing is recommended.

## 5 Limitations of Study

The in silico allergenicity prediction is based on a subset of known allergens profiled and reviewed in the search database (UniProtKB). This approach follows the WHO guidelines for preliminary allergenicity screening based on sequence homology prior to further testing (Mullins et al. 2022). While UniProtKB is recognized as an ongoing repository for the deposit of newly profiled proteins, novel or under‐characterized allergens from the valorized olive by‐products may lack representation at the time of the study. This study is a preliminary approach to generate initial insights prior to additional tests (e.g., de novo protein sequencing and clinical allergenicity trials).

## Author Contributions

**Joseph O'Shea:** methodology, investigation. **Catherine Barry‐Ryan:** conceptualization, supervision, funding acquisition, investigation, writing – review and editing, project administration. **Paz Otero:** methodology, investigation, funding acquisition, writing – original draft. **Antía Torres:** methodology, writing – original draft, investigation. **Maame Ekua Manful:** conceptualization, writing – original draft, methodology, investigation, writing – review and editing.

## Funding

This study was funded by the Bio Based Industries Joint Undertaking (JU) under grant agreement No 888003. The JU receives support from the European Union's Horizon 2020 research and innovation program and the Bio Based Industries Consortium.

## Conflicts of Interest

The authors declare no conflicts of interest.

## Supporting information


**Data S1:** Complete amino acid sequencing data for all three batches of the XOS extract.
**Table S1:** Amino acid sequences identified in Batch 2 (used for in silico analyses). Table includes the following information: Unique identifier assigned to the protein in the UniProtKB database (**Accession**); Description of the protein and its origin (**Description**); Percentage of the protein sequence that aligns with known proteins in the database (**Coverage [%]**); Number of amino acids in the protein sequence (**#Aas**); Amino acid sequence of the protein (**Sequence**); The function that the protein performs at the molecular level (**Molecular function**). Proteins identified in all three batches are indicated in bold.
**Table S2:** Amino acid sequences identified in Batch 1. Table includes the following information: Unique identifier assigned to the protein in the UniProtKB database (**Accession**); Description of the protein and its origin (**Description**); Percentage of the protein sequence that aligns with known proteins in the database (**Coverage [%]**); Number of amino acids in the protein sequence (**#Aas**); Amino acid sequence of the protein (**Sequence**); The function that the protein performs at the molecular level (**Molecular function**). Proteins identified in all three batches are indicated in bold.
**Table S3:** Amino acid sequences identified in Batch 3. Table includes the following information. Unique identifier assigned to the protein in the UniProtKB database (**Accession**); Description of the protein and its origin (**Description**); Percentage of the protein sequence that aligns with known proteins in the database (**Coverage [%]**); Number of amino acids in the protein sequence (**#Aas**); Amino acid sequence of the protein (**Sequence**); The function that the protein performs at the molecular level (**Molecular function**). Proteins identified in all three batches are indicated in bold.

**Data S2:** AllerCatPro 2.0 output for amino acid sequences 1–50 of the XOS extract.

**Data S3:** AllerCatPro 2.0 output for amino acid sequences 51–83 of the XOS extract.

## Data Availability

The data that supports the findings of this study are available in the Supporting Information of this article.
