# Supplementary material for: Assessment of Allergenic Potential for Cross‐Reactivity of Olive ( Olea europaea ) Pit Xylo‐Oligosaccharide‐Rich Extract With Legislated Allergens in the European Union
Source: Food Sci Nutr. 2026 Aug 3;14(8):e72207. doi: 10.1002/fsn3.72207 (PMC13434218; doi:10.1002/fsn3.72207)
Supplement: Supplementary file 1 [file FSN3-14-e72207-s002.pdf]

AllerCatPro 2.0 Results

Protein sequence of interest					Predicted most similar allergen (non-autoimmune allergens)		Potential cross-reactivity of query protein (# and links)	Protein info, functionality of most similar allergen				Clinical relevance of most similar allergen		Similarity to most similar allergen			Similarity to allergen and resulting predicted evidence for allergenicity		Similarity to autoimmune allergen (% identity to closest human hit)	Similarity to low allergenic protein (% identity to closest hit)
Query#	Name	Sequence Length	Gluten allergens (# of Q-repeats)	# of 3x6-mer overlaps	Protein allergen	Species		UniProt/NCBI	Functionality (SUPFAM)	Pfam	InterPro	IgE prevalence	Allergen info	% identity, linear 80 aa window	% identity, 3D epitope	Show 3D epitope	Result	Comment		
1	tr|A0A8S0PS52|A0A8S0PS52_OLEEU Actin-7 OS=Olea europaea subsp. europaea OX=158383 GN=OLEA9_A041632 P	377	0	-	No significant hit (similar to autoimmune allergen)	-	1	-	-	-	-	-	-	-	-	-	no evidence	no hits	88.1	-
2	tr|A0A8S0UQW6|A0A8S0UQW6_OLEEU Actin OS=Olea europaea subsp. europaea OX=158383 GN=OLEA9_A047380 PE=	377	0	-	No significant hit (similar to autoimmune allergen)	-	1	-	-	-	-	-	-	-	-	-	no evidence	no hits	87.0	-
3	tr|A0A8S0RRP2|A0A8S0RRP2_OLEEU Heat shock cognate 70 kDa 2-like OS=Olea europaea subsp. europaea OX=	650	0	-	Tyr p 28	Tyrophagus putrescentiae	15	A0A1B2YLJ2	Actin-like ATPase domain (SSF53067); Heat shock protein 70kD (HSP70), peptide-binding domain (SSF100920); Heat shock protein 70kD (HSP70), C-terminal subdomain (SSF100934)	PF00012	-	-	-	91.2	100.0	show in structure	strong evidence	3Depi >93%	-	-
4	tr|A0A8S0SNJ7|A0A8S0SNJ7_OLEEU phosphopyruvate hydratase OS=Olea europaea subsp. europaea OX=158383	469	0	-	Amb a 12	Ambrosia artemisiifolia	37	A0A1B2H9Q5	-	-	-	-	-	97.5	100.0	show in structure	strong evidence	3Depi >93%	-	-
5	tr|A0A8S0PCB3|A0A8S0PCB3_OLEEU Elongation factor 1-alpha OS=Olea europaea subsp. europaea OX=158383	483	0	-	No significant hit (E-value threshold 0.001)	-	0	-	-	-	-	-	-	-	-	-	no evidence	Similar to low allergenic protein	-	96.4
6	tr|A0A8S0SIJ5|A0A8S0SIJ5_OLEEU Heat shock 83 OS=Olea europaea subsp. europaea OX=158383 GN=OLEA9_A07	593	0	-	Hev b HSP80	Hevea brasiliensis	3	Q71EE1	HSP90 C-terminal domain (SSF110942); Ribosomal protein S5 domain 2-like (SSF54211); ATPase domain of HSP90 chaperone/DNA topoisomerase II/histidine kinase (SSF55874)	PF02518 PF00183	IPR003594 IPR036890 IPR019805 IPR037196 IPR001404 IPR020575 IPR020568	-	AG(1)	98.8	100.0	show in structure	strong evidence	3Depi >93%	-	-
7	tr|A0A8S0SJ95|A0A8S0SJ95_OLEEU Histone H2A OS=Olea europaea subsp. europaea OX=158383 GN=OLEA9_A0169	151	0	-	No significant hit (E-value threshold 0.001)	-	0	-	-	-	-	-	-	-	-	-	no evidence	no hits	-	81.8
8	tr|A0A8S0QCC2|A0A8S0QCC2_OLEEU GTPase Era OS=Olea europaea subsp. europaea OX=158383 GN=OLEA9_A04773	437	0	-	No significant hit (E-value threshold 0.001)	-	0	-	-	-	-	-	-	-	-	-	no evidence	no hits	-	-
9	tr|W1IA56|W1IA56_OLEEU E1 ubiquitin-activating enzyme (Fragment) OS=Olea europaea OX=4146 GN=ubiA PE	248	0	-	No significant hit (E-value threshold 0.001)	-	0	-	-	-	-	-	-	-	-	-	no evidence	no hits	-	-
10	tr|A0A8S0PBF6|A0A8S0PBF6_OLEEU Morc S5 domain-containing protein OS=Olea europaea subsp. europaea OX	779	0	-	No significant hit (E-value threshold 0.001)	-	0	-	-	-	-	-	-	-	-	-	no evidence	no hits	-	-
11	tr|A0A8S0QB37|A0A8S0QB37_OLEEU Heat shock 83 OS=Olea europaea subsp. europaea OX=158383 GN=OLEA9_A04	703	0	-	Hev b HSP80	Hevea brasiliensis	3	Q71EE1	HSP90 C-terminal domain (SSF110942); Ribosomal protein S5 domain 2-like (SSF54211); ATPase domain of HSP90 chaperone/DNA topoisomerase II/histidine kinase (SSF55874)	PF02518 PF00183	IPR003594 IPR036890 IPR019805 IPR037196 IPR001404 IPR020575 IPR020568	-	AG(1)	100.0	100.0	show in structure	strong evidence	3Depi >93%	-	-
12	tr|A0A8S0SQC9|A0A8S0SQC9_OLEEU Uncharacterized protein OS=Olea europaea subsp. europaea OX=158383 GN	245	0	-	No significant hit (E-value threshold 0.001)	-	0	-	-	-	-	-	-	-	-	-	no evidence	no hits	-	-
13	tr|A0A8S0QT36|A0A8S0QT36_OLEEU Cell division cycle 48 homolog OS=Olea europaea subsp. europaea OX=15	837	0	-	Katanin	Triticum aestivum	3	A0A7J8RNU5	-	-	-	-	-	53.8	-	-	strong evidence	win80lin >35%	-	-
14	tr|A0A8S0UVW6|A0A8S0UVW6_OLEEU inorganic diphosphatase OS=Olea europaea subsp. europaea OX=158383 GN	480	0	-	Der f 32	Dermatophagoides farinae	2	A0A088SCP3	Inorganic pyrophosphatase (SSF50324)	PF00719	-	-	AG(2)	38.8	-	-	strong evidence	win80lin >35%	-	29.7
15	tr|A0A8S0RUZ6|A0A8S0RUZ6_OLEEU UDP-glycosyltransferase 83A1-like OS=Olea europaea subsp. europaea OX	461	0	-	No significant hit (E-value threshold 0.001)	-	0	-	-	-	-	-	-	-	-	-	no evidence	no hits	-	-
16	tr|W1I6H3|W1I6H3_OLEEU Heterogeneous nuclear ribonucleoprotein H2 (Fragment) OS=Olea europaea OX=414	221	0	-	No significant hit (E-value threshold 0.001)	-	0	-	-	-	-	-	-	-	-	-	no evidence	no hits	-	-
17	tr|A0A8S0R4B0|A0A8S0R4B0_OLEEU Fructose-bisphosphate aldolase OS=Olea europaea subsp. europaea OX=15	689	0	-	Pan h 3	Pangasianodon hypophthalmus	9	UPI000F00FADE	-	PF00274	-	-	-	77.5	93.3	show in structure	strong evidence	3Depi >93%	-	-
18	tr|A0A8S0QBD8|A0A8S0QBD8_OLEEU Proteasome subunit alpha type OS=Olea europaea subsp. europaea OX=158	249	0	-	No significant hit (E-value threshold 0.001)	-	0	-	-	-	-	-	-	-	-	-	no evidence	no hits	-	-
19	tr|A0A8S0SWP6|A0A8S0SWP6_OLEEU Tubulin beta chain OS=Olea europaea subsp. europaea OX=158383 GN=OLEA	539	0	-	Lep d 33	Lepidoglyphus destructor	6	Q8WQ47	Tubulin nucleotide-binding domain-like (SSF52490); Tubulin C-terminal domain-like (SSF55307)	PF00091 PF03953	IPR000217 IPR002452 IPR003008 IPR008280 IPR017975 IPR018316 IPR023123 IPR036525 IPR037103	95	AG(2)	55.0	76.9	show in structure	weak evidence	3Depi <=93%	41.0	-
20	tr|A0A8S0REP5|A0A8S0REP5_OLEEU Heat shock 90-5, chloroplastic-like OS=Olea europaea subsp. europaea	794	0	-	Hev b HSP80	Hevea brasiliensis	3	Q71EE1	HSP90 C-terminal domain (SSF110942); Ribosomal protein S5 domain 2-like (SSF54211); ATPase domain of HSP90 chaperone/DNA topoisomerase II/histidine kinase (SSF55874)	PF02518 PF00183	IPR003594 IPR036890 IPR019805 IPR037196 IPR001404 IPR020575 IPR020568	-	AG(1)	65.0	81.2	show in structure	weak evidence	3Depi <=93%	-	-
21	tr|A0A8S0ST76|A0A8S0ST76_OLEEU Histone H4 OS=Olea europaea subsp. europaea OX=158383 GN=OLEA9_A08397	184	0	-	No significant hit (E-value threshold 0.001)	-	0	-	-	-	-	-	-	-	-	-	no evidence	no hits	-	87.6

22	tr|A0A8S0V1P6|A0A8S0V1P6_OLEEU BZIP transcription factor 11-like OS=Olea europaea subsp. europaea OX	135	0	-	No significant hit (E-value threshold 0.001)	-	0	-	-	-	-	-	-	-	-	-	no evidence	no hits	-	-
23	tr|A0A8S0ULM7|A0A8S0ULM7_OLEEU 40S ribosomal S28-2 OS=Olea europaea subsp. europaea OX=158383 GN=OLE	124	0	-	No significant hit (E-value threshold 0.001)	-	0	-	-	-	-	-	-	-	-	-	no evidence	no hits	-	-
24	tr|A0A8S0UYU6|A0A8S0UYU6_OLEEU ATP synthase subunit beta OS=Olea europaea subsp. europaea OX=158383	627	0	-	ATP synthase subunit beta, mitochondrial	Penicillium glabrum	1	P85446	P-loop containing nucleoside triphosphate hydrolases (SSF52540)	-	-	-	-	91.5	-	-	strong evidence	win80lin >35%	-	29.0
25	tr|A0A8S0SZP0|A0A8S0SZP0_OLEEU tryptophan synthase OS=Olea europaea subsp. europaea OX=158383 GN=OLE	264	0	-	No significant hit (E-value threshold 0.001)	-	0	-	-	-	-	-	-	-	-	-	no evidence	no hits	-	63.2
26	tr|A0A8S0UR70|A0A8S0UR70_OLEEU dolichol kinase OS=Olea europaea subsp. europaea OX=158383 GN=OLEA9_A	602	0	-	No significant hit (E-value threshold 0.001)	-	0	-	-	-	-	-	-	-	-	-	no evidence	no hits	-	-
27	tr|A0A8S0V5Y0|A0A8S0V5Y0_OLEEU Probable (S)-N-methylcoclaurine 3 -hydroxylase isozyme 2 OS=Olea euro	498	0	-	No significant hit (E-value threshold 0.001)	-	0	-	-	-	-	-	-	-	-	-	no evidence	no hits	-	-
28	tr|A0A8S0UH33|A0A8S0UH33_OLEEU Uncharacterized protein OS=Olea europaea subsp. europaea OX=158383 GN	195	0	-	No significant hit (E-value threshold 0.001)	-	0	-	-	-	-	-	-	-	-	-	no evidence	no hits	-	-
29	tr|A0A8S0SLP5|A0A8S0SLP5_OLEEU BON1-associated 2-like OS=Olea europaea subsp. europaea OX=158383 GN=	171	0	-	No significant hit (E-value threshold 0.001)	-	0	-	-	-	-	-	-	-	-	-	no evidence	no hits	-	-
30	tr|A0A8S0PEB4|A0A8S0PEB4_OLEEU LRR receptor-like serine threonine- kinase GSO1 OS=Olea europaea subs	645	0	-	Tri a 23kd	Triticum aestivum	1	Q4W1G0	-	-	-	-	-	38.0	-	-	strong evidence	win80lin >35%	-	-
31	tr|A0A8S0TH01|A0A8S0TH01_OLEEU Disease resistance At4g27190-like (Fragment) OS=Olea europaea subsp.	295	0	-	No significant hit (E-value threshold 0.001)	-	0	-	-	-	-	-	-	-	-	-	no evidence	no hits	-	-
32	tr|A0A8S0TI61|A0A8S0TI61_OLEEU Zinc finger ZAT10-like OS=Olea europaea subsp. europaea OX=158383 GN=	278	0	-	No significant hit (E-value threshold 0.001)	-	0	-	-	-	-	-	-	-	-	-	no evidence	no hits	-	-
33	tr|A0A8S0QDK2|A0A8S0QDK2_OLEEU RNA helicase OS=Olea europaea subsp. europaea OX=158383 GN=OLEA9_A004	443	0	-	No significant hit (E-value threshold 0.001)	-	0	-	-	-	-	-	-	-	-	-	no evidence	Similar to low allergenic protein	-	90.3
34	tr|A0A8S0VDN6|A0A8S0VDN6_OLEEU Elongation factor 2 OS=Olea europaea subsp. europaea OX=158383 GN=OLE	1185	0	-	Der f EF	Dermatophagoides farinae	1	L7UW58	Translation proteins (SSF50447); Ribosomal protein S5 domain 2-like (SSF54211); EF-G C-terminal domain-like (SSF54980)	PF00679 PF14492 PF03764	IPR041095 IPR035647 IPR000640 IPR020568 IPR014721 IPR009000 IPR005517	-	AG(1)	78.8	-	-	strong evidence	win80lin >35%. Similar to low allergenic protein	-	93.8
35	tr|A0A8S0QE66|A0A8S0QE66_OLEEU 40S ribosomal protein S8 OS=Olea europaea subsp. europaea OX=158383 G	268	0	-	No significant hit (E-value threshold 0.001)	-	0	-	-	-	-	-	-	-	-	-	no evidence	no hits	-	-
36	tr|A0A8S0TEN5|A0A8S0TEN5_OLEEU 40S ribosomal S3-3 OS=Olea europaea subsp. europaea OX=158383 GN=OLEA	301	0	-	Asp f RPS3	Neosartorya fumigata	2	B0XP55	Prokaryotic type KH domain (KH-domain type II) (SSF54814); Ribosomal protein S3 C-terminal domain (SSF54821)	PF07650 PF00189	IPR015946 IPR004044 IPR009019 IPR001351 IPR036419 IPR018280 IPR005703	-	AG(1)	86.2	92.9	show in structure	weak evidence	3Depi <=93%	-	-
37	tr|A0A8S0SRB4|A0A8S0SRB4_OLEEU Uncharacterized protein OS=Olea europaea subsp. europaea OX=158383 GN	1142	0	-	No significant hit (E-value threshold 0.001)	-	0	-	-	-	-	-	-	-	-	-	no evidence	no hits	-	-
38	tr|A0A8S0SYQ1|A0A8S0SYQ1_OLEEU Inactive ubiquitin carboxyl-terminal hydrolase 54 OS=Olea europaea su	1629	0	-	No significant hit (E-value threshold 0.001)	-	0	-	-	-	-	-	-	-	-	-	no evidence	no hits	-	-
39	tr|A0A8S0PJZ8|A0A8S0PJZ8_OLEEU 60S ribosomal L12 OS=Olea europaea subsp. europaea OX=158383 GN=OLEA9	166	0	-	No significant hit (E-value threshold 0.001)	-	0	-	-	-	-	-	-	-	-	-	no evidence	Similar to low allergenic protein	-	92.8
40	tr|A0A8S0RX51|A0A8S0RX51_OLEEU Histone-lysine N-methyltransferase, H3 lysine-9 specific SUVH1-like O	724	0	-	No significant hit (E-value threshold 0.001)	-	0	-	-	-	-	-	-	-	-	-	no evidence	no hits	-	-
41	tr|A0A8S0PEI1|A0A8S0PEI1_OLEEU Pentatricopeptide repeat-containing At5g46100 OS=Olea europaea subsp.	480	0	-	No significant hit (E-value threshold 0.001)	-	0	-	-	-	-	-	-	-	-	-	no evidence	no hits	-	-
42	tr|A0A8S0U7Q4|A0A8S0U7Q4_OLEEU Uncharacterized protein OS=Olea europaea subsp. europaea OX=158383 GN	110	0	-	No significant hit (E-value threshold 0.001)	-	0	-	-	-	-	-	-	-	-	-	no evidence	no hits	-	-
43	tr|A0A8S0RVC3|A0A8S0RVC3_OLEEU Aldehyde dehydrogenase OS=Olea europaea subsp. europaea OX=158383 GN=	499	0	-	Cla h 10	Cladosporium herbarum	6	P40108	ALDH-like (SSF53720)	PF00171	-	-	-	50.0	61.5	show in structure	weak evidence	3Depi <=93%	-	-
44	tr|A0A8S0PR25|A0A8S0PR25_OLEEU DUF674 domain-containing protein OS=Olea europaea subsp. europaea OX=	241	0	-	No significant hit (E-value threshold 0.001)	-	0	-	-	-	-	-	-	-	-	-	no evidence	no hits	-	-
45	tr|A0A8S0QIX0|A0A8S0QIX0_OLEEU Transcription factor bHLH18-like OS=Olea europaea subsp. europaea OX=	348	0	-	No significant hit (E-value threshold 0.001)	-	0	-	-	-	-	-	-	-	-	-	no evidence	no hits	-	-
46	tr|A0A8S0V0B1|A0A8S0V0B1_OLEEU Calcium uniporter protein OS=Olea europaea subsp. europaea OX=158383	345	0	-	No significant hit (E-value threshold 0.001)	-	0	-	-	-	-	-	-	-	-	-	no evidence	no hits	-	-
47	tr|A0A8S0PMV2|A0A8S0PMV2_OLEEU Uncharacterized protein OS=Olea europaea subsp. europaea OX=158383 GN	504	0	-	No significant hit (E-value threshold 0.001)	-	0	-	-	-	-	-	-	-	-	-	no evidence	no hits	-	-
48	tr|A0A8S0TWA0|A0A8S0TWA0_OLEEU Filament-like plant protein OS=Olea europaea subsp. europaea OX=15838	659	0	-	No significant hit (E-value threshold 0.001)	-	0	-	-	-	-	-	-	-	-	-	no evidence	no hits	-	-
49	tr|A0A8S0S8S6|A0A8S0S8S6_OLEEU DNA-directed RNA polymerase OS=Olea europaea subsp. europaea OX=15838	1038	0	-	No significant hit (E-value threshold 0.001)	-	0	-	-	-	-	-	-	-	-	-	no evidence	no hits	-	-
50	tr|A0A8S0SET3|A0A8S0SET3_OLEEU DUF4005 domain-containing protein OS=Olea europaea subsp. europaea OX	188	0	-	No significant hit (E-value threshold 0.001)	-	0	-	-	-	-	-	-	-	-	-	no evidence	no hits	-	-

Limit for webserver analysis reached (50 proteins). Please connect with us for running larger queries.	

Download result as comma-separated table (can also be opened in Excel)

Summary result (Model 2.0 cv 1.1):	
strong evidence	10/50 (20.0%)
weak evidence	4/50 (8.0%)
no evidence	36/50 (72.0%)
no hits	33
3Depi >93%	5
win80lin >35%	4
3Depi <=93%	4
Similar to low allergenic protein	3
win80lin >35%. Similar to low allergenic protein	1

A*STAR Bioinformatics Institute
P&G Global Product Stewardship
