# Supplementary material for: Assessment of Allergenic Potential for Cross‐Reactivity of Olive ( Olea europaea ) Pit Xylo‐Oligosaccharide‐Rich Extract With Legislated Allergens in the European Union
Source: Food Sci Nutr. 2026 Aug 3;14(8):e72207. doi: 10.1002/fsn3.72207 (PMC13434218; doi:10.1002/fsn3.72207)
Supplement: Supplementary file 2 [file FSN3-14-e72207-s003.pdf]

AllerCatPro 2.0 Results

Protein sequence of interest					Predicted most similar allergen (non-autoimmune allergens)		Potential cross-reactivity of query protein (# and links)	Protein info, functionality of most similar allergen				Clinical relevance of most similar allergen		Similarity to most similar allergen			Similarity to allergen and resulting predicted evidence for allergenicity		Similarity to autoimmune allergen (% identity to closest human hit)	Similarity to low allergenic protein (% identity to closest hit)
Query#	Name	Sequence Length	Gluten allergens (# of Q-repeats)	# of 3x6-mer overlaps	Protein allergen	Species		UniProt/NCBI	Functionality (SUPFAM)	Pfam	InterPro	IgE prevalence	Allergen info	% identity, linear 80 aa window	% identity, 3D epitope	Show 3D epitope	Result	Comment		
1	tr|A0A8S0UEF5|A0A8S0UEF5_OLEEU Structural maintenance of chromosomes protein OS=Olea europaea subsp.	1247	0	-	No significant hit (E-value threshold 0.001)	-	0	-	-	-	-	-	-	-	-	-	no evidence	no hits	-	-
2	tr|A0A8S0RLA4|A0A8S0RLA4_OLEEU Beta-glucuronosyltransferase 14A-like OS=Olea europaea subsp. europae	435	0	-	No significant hit (E-value threshold 0.001)	-	0	-	-	-	-	-	-	-	-	-	no evidence	no hits	-	-
3	tr|A0A8S0TQ48|A0A8S0TQ48_OLEEU Probable fructokinase-7 OS=Olea europaea subsp. europaea OX=158383 GN	347	0	-	No significant hit (E-value threshold 0.001)	-	0	-	-	-	-	-	-	-	-	-	no evidence	no hits	-	59.9
4	tr|A0A8S0R5S2|A0A8S0R5S2_OLEEU BRANCHLESS TRICHOME OS=Olea europaea subsp. europaea OX=158383 GN=OLE	322	0	-	No significant hit (E-value threshold 0.001)	-	0	-	-	-	-	-	-	-	-	-	no evidence	no hits	-	-
5	tr|A0A8S0RUX3|A0A8S0RUX3_OLEEU PREDICTED: uncharacterized protein LOC105974453 OS=Olea europaea subs	178	0	-	No significant hit (E-value threshold 0.001)	-	0	-	-	-	-	-	-	-	-	-	no evidence	no hits	-	-
6	tr|A0A8S0QK29|A0A8S0QK29_OLEEU 14-3-3 A OS=Olea europaea subsp. europaea OX=158383 GN=OLEA9_A003128	327	0	-	No significant hit (E-value threshold 0.001)	-	0	-	-	-	-	-	-	-	-	-	no evidence	no hits	-	81.2
7	tr|A0A8S0RDK1|A0A8S0RDK1_OLEEU Zinc finger BED domain-containing RICESLEEPER 2-like OS=Olea europaea	211	0	-	No significant hit (E-value threshold 0.001)	-	0	-	-	-	-	-	-	-	-	-	no evidence	no hits	-	-
8	tr|A0A8S0TXF9|A0A8S0TXF9_OLEEU ADP-ribosylation factor OS=Olea europaea subsp. europaea OX=158383 GN	224	0	0	No significant hit	-	1	-	-	-	-	-	-	-	-	-	no evidence	no hits	-	30.6
9	tr|A0A8S0PHV1|A0A8S0PHV1_OLEEU Arogenate dehydratase OS=Olea europaea subsp. europaea OX=158383 GN=O	420	0	-	No significant hit (E-value threshold 0.001)	-	0	-	-	-	-	-	-	-	-	-	no evidence	no hits	-	-
10	tr|A0A8S0SNF1|A0A8S0SNF1_OLEEU Uncharacterized protein (Fragment) OS=Olea europaea subsp. europaea O	162	0	-	No significant hit	-	2	-	-	-	-	-	-	-	-	-	no evidence	no hits	-	-
11	tr|A0A8S0R6G9|A0A8S0R6G9_OLEEU Kinesin-like protein OS=Olea europaea subsp. europaea OX=158383 GN=OL	657	0	-	No significant hit (E-value threshold 0.001)	-	0	-	-	-	-	-	-	-	-	-	no evidence	no hits	-	-
12	tr|A0A8S0TKL8|A0A8S0TKL8_OLEEU 40S ribosomal S9 OS=Olea europaea subsp. europaea OX=158383 GN=OLEA9_	195	0	-	No significant hit (E-value threshold 0.001)	-	0	-	-	-	-	-	-	-	-	-	no evidence	no hits	-	69.9
13	tr|A0A2K9RSI5|A0A2K9RSI5_9LAMI DNA-directed RNA polymerase subunit beta'' OS=Olea exasperata OX=4957	1394	0	-	No significant hit (E-value threshold 0.001)	-	0	-	-	-	-	-	-	-	-	-	no evidence	no hits	-	-
14	tr|A0A8S0VHD2|A0A8S0VHD2_OLEEU SRG1-like OS=Olea europaea subsp. europaea OX=158383 GN=OLEA9_A050069	350	0	-	No significant hit (E-value threshold 0.001)	-	0	-	-	-	-	-	-	-	-	-	no evidence	no hits	-	30.3
15	tr|A0A8S0UX23|A0A8S0UX23_OLEEU Endo-1,3 1,4-beta-D-glucanase-like OS=Olea europaea subsp. europaea O	241	0	-	No significant hit (E-value threshold 0.001)	-	0	-	-	-	-	-	-	-	-	-	no evidence	no hits	-	-
16	tr|A0A8S0QEF9|A0A8S0QEF9_OLEEU (S)-ureidoglycine aminohydrolase OS=Olea europaea subsp. europaea OX=	76	0	0	No significant hit (E-value threshold 0.001)	-	0	-	-	-	-	-	-	-	-	-	no evidence	no hits	-	-
17	tr|A0A8S0RWP0|A0A8S0RWP0_OLEEU Catalase isozyme 1 OS=Olea europaea subsp. europaea OX=158383 GN=OLEA	296	0	-	No significant hit (E-value threshold 0.001)	-	0	-	-	-	-	-	-	-	-	-	no evidence	no hits	-	-
18	tr|A0A8S0Q8A0|A0A8S0Q8A0_OLEEU Zinc finger ZAT4-like OS=Olea europaea subsp. europaea OX=158383 GN=O	500	0	-	No significant hit (E-value threshold 0.001)	-	0	-	-	-	-	-	-	-	-	-	no evidence	no hits	-	-
19	tr|A0A8S0RRT2|A0A8S0RRT2_OLEEU IQ-DOMAIN 1 OS=Olea europaea subsp. europaea OX=158383 GN=OLEA9_A0643	313	0	-	No significant hit (E-value threshold 0.001)	-	0	-	-	-	-	-	-	-	-	-	no evidence	no hits	-	-
20	tr|A0A8S0PP20|A0A8S0PP20_OLEEU LEAF RUST 10 DISEASE-RESISTANCE LOCUS RECEPTOR-LIKE PROTEIN KINASE-li	607	0	-	Unknown	Oryza sativa	3	AK068307	-	-	-	-	-	46.2	64.7	show in structure	weak evidence	3Depi <=93%	-	-
21	tr|A0A8S0T4M7|A0A8S0T4M7_OLEEU Filament-like plant protein 3 OS=Olea europaea subsp. europaea OX=158	589	0	-	No significant hit (E-value threshold 0.001)	-	0	-	-	-	-	-	-	-	-	-	no evidence	no hits	-	-
22	tr|A0A8S0PFE4|A0A8S0PFE4_OLEEU Dihydrolipoyl dehydrogenase OS=Olea europaea subsp. europaea OX=15838	542	0	-	No significant hit (E-value threshold 0.001)	-	0	-	-	-	-	-	-	-	-	-	no evidence	no hits	-	34.8
23	tr|A0A8S0QI47|A0A8S0QI47_OLEEU Uncharacterized oxidoreductase OS=Olea europaea subsp. europaea OX=15	359	0	-	No significant hit (E-value threshold 0.001)	-	0	-	-	-	-	-	-	-	-	-	no evidence	no hits	-	-
24	tr|A0A8S0UP35|A0A8S0UP35_OLEEU 9-cis-epoxycarotenoid dioxygenase NCED6, chloroplastic OS=Olea europa	570	0	-	No significant hit (E-value threshold 0.001)	-	0	-	-	-	-	-	-	-	-	-	no evidence	no hits	-	-
25	tr|A0A8S0UVB2|A0A8S0UVB2_OLEEU ABC transporter B family member 2-like OS=Olea europaea subsp. europa	1336	0	-	No significant hit (E-value	-	0	-	-	-	-	-	-	-	-	-	no evidence	no hits	-	-

					threshold 0.001)															
26	tr|A0A8S0R0X1|A0A8S0R0X1_OLEEU Uncharacterized protein OS=Olea europaea subsp. europaea OX=158383 GN	269	0	-	No significant hit (E-value threshold 0.001)	-	0	-	-	-	-	-	-	-	-	-	no evidence	no hits	-	-
27	tr|A0A8S0UEA9|A0A8S0UEA9_OLEEU Uncharacterized protein OS=Olea europaea subsp. europaea OX=158383 GN	612	0	-	No significant hit (E-value threshold 0.001)	-	0	-	-	-	-	-	-	-	-	-	no evidence	no hits	-	-
28	tr|A0A8S0UYB9|A0A8S0UYB9_OLEEU Phosphoinositide phospholipase C OS=Olea europaea subsp. europaea OX=	548	0	-	No significant hit (E-value threshold 0.001)	-	0	-	-	-	-	-	-	-	-	-	no evidence	no hits	-	-
29	tr|A0A8S0QI89|A0A8S0QI89_OLEEU Diphthamide biosynthesis protein 3 OS=Olea europaea subsp. europaea O	80	0	-	No significant hit (E-value threshold 0.001)	-	0	-	-	-	-	-	-	-	-	-	no evidence	no hits	-	-
30	tr|A0A8S0RR81|A0A8S0RR81_OLEEU AAA+ ATPase domain-containing protein OS=Olea europaea subsp. europae	949	0	-	No significant hit (E-value threshold 0.001)	-	0	-	-	-	-	-	-	-	-	-	no evidence	no hits	-	-
31	tr|A0A8S0SB03|A0A8S0SB03_OLEEU Helicase MAGATAMA 3 OS=Olea europaea subsp. europaea OX=158383 GN=OLE	1104	0	-	No significant hit (E-value threshold 0.001)	-	0	-	-	-	-	-	-	-	-	-	no evidence	no hits	-	-
32	tr|A0A8S0TEC5|A0A8S0TEC5_OLEEU Heat shock cognate 70 kDa 2-like OS=Olea europaea subsp. europaea OX=	679	0	-	Tyr p 28	Tyrophagus putrescentiae	15	A0A1B2YLJ2	Actin-like ATPase domain (SSF53067); Heat shock protein 70kD (HSP70), peptide-binding domain (SSF100920); Heat shock protein 70kD (HSP70), C-terminal subdomain (SSF100934)	PF00012	-	-	-	92.5	100.0	show in structure	strong evidence	3Depi >93%	-	-
33	tr|A0A8S0PK37|A0A8S0PK37_OLEEU poly(A)-specific ribonuclease OS=Olea europaea subsp. europaea OX=158	274	0	-	No significant hit (E-value threshold 0.001)	-	0	-	-	-	-	-	-	-	-	-	no evidence	no hits	-	-

Download result as comma-separated table (can also be opened in Excel)

Summary result (Model 2.0 cv 1.1):	
strong evidence	1/33 (3.0%)
weak evidence	1/33 (3.0%)
no evidence	31/33 (93.9%)
no hits	31
3Depi <=93%	1
3Depi >93%	1

Bioinformatics Institute

A*STAR

Global Product Stewardship
