# Supplementary material for: Assessment of Allergenic Potential for Cross‐Reactivity of Olive ( Olea europaea ) Pit Xylo‐Oligosaccharide‐Rich Extract With Legislated Allergens in the European Union
Source: Food Sci Nutr. 2026 Aug 3;14(8):e72207. doi: 10.1002/fsn3.72207 (PMC13434218; doi:10.1002/fsn3.72207)
Supplement: Supplementary file 3 [file FSN3-14-e72207-s001.docx]

**Table S1.** Amino acid sequences identified in Batch 2 (used for in silico analyses). Table includes the following information: Unique identifier assigned to the protein in the UniProtKB database (**Accession**); Description of the protein and its origin (**Description**); Percentage of the protein sequence that aligns with known proteins in the database (**Coverage [%]**; Number of amino acids in the protein sequence (**#Aas);**  Amino acid sequence of the protein (**Sequence**); The function that the protein performs at the molecular level (**Molecular function**). Proteins identified in all three batches are indicated in **bold**.

| **Accession** | **Description** | **Coverage [%]** | **# AAs** | **Sequence** | **Molecular Function** |
| --- | --- | --- | --- | --- | --- |
| **XOS Batch 2** | | | | | |
| **A0A8S0PS52** | **submitted name: Actin-7 [OS=Olea europaea subsp. europaea]** | **14** | **377** | **VAPEEHPVLLTEAPLNPK; IWHHTFYNELR;AGFAGDDAPR;DLTDYLMK;RGILTLK** | **ATP binding; Hydrolase activity** |
| **A0A8S0SJ95** | **submitted name: Actin [OS=Olea europaea subsp. europaea]** | **10** | **377** | **IWHHTFYNELR; GYSFTTTAER; AGFAGDDAPR; RGILTLK** | **ATP binding; Hydrolase activity** |
| A0A8S0RRP2 | submitted name: Heat shock cognate 70 kDa 2-like [OS=Olea europaea subsp. europaea] | 7 | 650 | IINEPTAAAIAYGLDK; VEIIANDQGNR; STAGDTHLGGEDFDNR | ATP binding; ATP-dependent protein folding chaperone |
| A0A8S0SNJ7 | phosphopyruvate hydratase [OS=Olea europaea subsp. europaea] | 5 | 469 | AAUPSGASTGIYEALELR; YNQLLR | Magnesium ion binding; Phosphopyruvate hydratase activity |
| **A0A8S0PCB3** | **Elongation factor 1-alpha [OS=Olea europaea subsp. europaea]** | **6** | **483** | **IGGIGTVPVGR; LPLQDVYK; STTTGHLIYK** | **Nucleic acid binding activity; GTPase activity; Translation elongation factor activity** |
| A0A8S0SIJ5 | submitted name: Heat shock 83 [OS=Olea europaea subsp. europaea] | 2 | 593 | TLTIIDSGIGMTK | ATP binding; ATP hydrolysis activity; ATP-dependent protein folding chaperone; Unfolded protein binding |
| **A0A8S0SJ95** | **Histone H2A [OS=Olea europaea subsp. europaea]** | **11** | **151** | **HLQLAIR; AGLQFPVGR** | **Nucleic acid binding activity; Protein heterodimerization activity; Structural constituent of chromatin** |
| **A0A8S0QCC2** | **submitted name: GTPase Era [OS=Olea europaea subsp. europaea]** | **2** | **437** | **KLEWYEK** | **Nucleic acid binding activity** |
| W1IA56 | submitted name: Putative Ubiquitin-activating enzyme E1 2 [OS=Olea europaea] | 6 | 248 | LAGTQPLEVLEAVQR | Ubiquitin-like modifier activating enzyme activity |
| A0A8S0PBF6 | Morc S5 domain-containing protein [OS=Olea europaea subsp. Europaea] | 2 | 779 | NSVSPVKETPATGK | ATP hydrolysis activity; Endonuclease activity |
| A0A8S0QB37 | submitted name: Heat shock 83 [OS=Olea europaea subsp. europaea] | 4 | 703 | ELISNASDALDK; SLTNDWEDHLAVK | ATP binding; ATP hydrolysis activity; ATP-dependent protein folding chaperone; Unfolded protein binding |
| A0A8S0SQC9 | submitted name: Uncharacterized protein [OS=Olea europaea subsp. europaea] | 5 | 245 | THASAYASINGIR | - |
| A0A8S0QT36 | submitted name: Cell division cycle 48 homolog [OS=Olea europaea subsp. europaea] | 2 | 837 | IVSQLLTLMDGLK | ATP binding; ATP hydrolysis activity |
| A0A8S0UVW6 | inorganic diphosphatase [OS=Olea europaea subsp. europaea] | 5 | 480 | LQIWDTAGQER; IILLGDSGVGK | GTP binding; GTPase activity; Inorganic diphosphatase phosphatase activity; Magnesium ion binding |
| A0A8S0RUZ6 | submitted name: UDP-glycosyltransferase 83A1-like [OS=Olea europaea subsp. europaea] | 2 | 461 | NKLEQLLTDER | UDP-glycosyltransferase activity |
| W1I6H3 | submitted name: Putative heterogeneous nuclear ribonucleoprotein F-like [OS=Olea europaea] | 8 | 221 | STGEAFVQFASQEIAEK | Nucleic acid binding activity |
| **A0A8S0R4B0** | **Fructose-bisphosphate aldolase [OS=Olea europaea subsp. europaea]** | **2** | **689** | **GILAADESTGTIGK** | **Fructose-bisphosphate aldolase activity** |
| **A0A8S0QBD8** | **Proteasome subunit alpha type [OS=Olea europaea subsp. europaea]** | **6** | **249** | **LTVEDPVTVEYITR** | **-** |
| A0A8S0SWP6 | Tubulin beta chain [OS=Olea europaea subsp. europaea] | 3 | 539 | NSSYFVEWIPNNVK | Cytoeskeletal activity; GTP binding; GTPase activity |
| A0A8S0REP5 | submitted name: Heat shock 90-5, chloroplastic-like [OS=Olea europaea subsp. europaea] | 2 | 794 | GVVDSDDLPLNVSR | ATP binding; ATP hydrolysis activity; ATP-dependent protein folding chaperone; Unfolded protein binding |
| **A0A8S0ST76** | **Histone H4 [OS=Olea europaea subsp. europaea]** | **5** | **184** | **ISGLIYEETR** | **Nucleic acid binding activity; Protein heterodimerization activity; Structural constituent of chromatin** |
| **A0A8S0V1P6** | **submitted name: BZIP transcription factor 11-like [OS=Olea europaea subsp. europaea]** | **9** | **135** | **LDDLSAQIAQLR** | **DNA-binding transcription factor activity; Protein heterodimerization activity** |
| A0A8S0ULM7 | submitted name: 40S ribosomal S28-2 [OS=Olea europaea subsp. europaea] | 10 | 124 | EGDVLTLLESER | Structural constituent of ribosome |
| A0A8S0UYU6 | ATP synthase subunit beta [OS=Olea europaea subsp. europaea] | 2 | 627 | IGLFGGAGVGK | Transporter activity; ATP binding; ATP hydrolysis activity |
| **A0A8S0SZP0** | **tryptophan synthase [OS=Olea europaea subsp. europaea]** | **3** | **264** | **ESTLHLVLR** | **mRNA binding; Structural constituent of ribosome; Tryptophan synthase activity** |
| **A0A8S0UR70** | **dolichol kinase [OS=Olea europaea subsp. europaea]** | **2** | **602** | **MAPLLLQLNGER** | **Kinase activity** |
| A0A8S0V5Y0 | submitted name: Probable (S)-N-methylcoclaurine 3 -hydroxylase isozyme 2 [OS=Olea europaea subsp. europaea] | 2 | 498 | ALDLQAEIR | Heme binding; Iron ion binding; Monooxygenase activity; Oxidoreductase activity |
| A0A8S0UH33 | submitted name: Uncharacterized protein [OS=Olea europaea subsp. europaea] | 6 | 195 | LNLDSNVEVQIR | - |
| A0A8S0SLP5 | submitted name: BON1-associated 2-like [OS=Olea europaea subsp. europaea] | 12 | 171 | MEKSSSRMIEITVISCEDLR | - |
| A0A8S0PEB4 | submitted name: LRR receptor-like serine threonine- kinase GSO1 [OS=Olea europaea subsp. europaea] | 2 | 645 | LDGSIPTSLGNLR | Kinase activity |
| A0A8S0TH01 | submitted name: Disease resistance At4g27190-like [OS=Olea europaea subsp. europaea] | 5 | 295 | TVEHIPGPSIMSQK | ADP binding |
| A0A8S0TI61 | submitted name: Zinc finger ZAT10-like [OS=Olea europaea subsp. europaea] | 9 | 278 | SGGDDQSTNSTTLSATTSGGGGSGR | DNA-binding transcription factor activity |
| A0A8S0QDK2 | ATP-dependent RNA helicase [OS=Olea europaea subsp. europaea] | 2 | 443 | VLITTDLLAR | Nucleic acid binding activity; ATP binding; Hydrolase activity; RNA helicase activity; Translation initiation factor activity |
| A0A8S0VDN6 | submitted name: Elongation factor 2 [OS=Olea europaea subsp. europaea] | 1 | 1185 | GVQYLNEIK | Nucleic acid binding activity; GTPase activity; Translation elongation factor activity |
| A0A8S0QE66 | 40S ribosomal protein S8 [OS=Olea europaea subsp. europaea] | 6 | 268 | ILDVVYNASNNELVR | Structural constituent of ribosome |
| A0A8S0TEN5 | submitted name: 40S ribosomal S3-3 [OS=Olea europaea subsp. europaea] | 3 | 301 | LLGGLAVR | Nucleic acid binding activity; Structural constituent of ribosome |
| A0A8S0SRB4 | submitted name: Uncharacterized protein [OS=Olea europaea subsp. europaea] | 1 | 1142 | DLFVADGEGLPR | - |
| A0A8S0SYQ1 | submitted name: Inactive ubiquitin carboxyl-terminal hydrolase 54 [OS=Olea europaea subsp. europaea] | 1 | 1629 | LLDSGFGLGQRVAERR | Cysteine-type deubiquitinase activity |
| A0A8S0PJZ8 | submitted name: 60S ribosomal L12 [OS=Olea europaea subsp. europaea] | 5 | 166 | IGPLGLSPK | Structural constituent of ribosome |
| A0A8S0RX51 | submitted name: Histone-lysine N-methyltransferase, H3 lysine-9 specific SUVH1-like [OS=Olea europaea subsp. europaea] | 2 | 724 | LTQLEEVKDMSPGVSR | Histone methyltransferase activity; Zinc ion binding |
| A0A8S0PEI1 | submitted name: Pentatricopeptide repeat-containing At5g46100 [OS=Olea europaea subsp. europaea] | 3 | 480 | LNDAVEILDRMK | - |
| A0A8S0U7Q4 | submitted name: Uncharacterized protein [OS=Olea europaea subsp. europaea] | 6 | 110 | IGFDLFK | - |
| A0A8S0RVC3 | Aldehyde dehydrogenase [OS=Olea europaea subsp. europaea] | 2 | 499 | HLTPVTLELGGK | Oxidoreductase activity |
| A0A8S0PR25 | DUF674 domain-containing protein [OS=Olea europaea subsp. europaea] | 3 | 241 | ELAALQEK | - |
| **A0A8S0QIX0** | **submitted name: Transcription factor bHLH18-like [OS=Olea europaea subsp. europaea]** | **3** | **348** | **FSDKDVLIR** | **Protein dimerization activity** |
| A0A8S0V0B1 | Calcium uniporter protein [OS=Olea europaea subsp. europaea] | 3 | 345 | KELDQLELQK | Transporter activity; Calcium channel activity; Uniporter activity |
| **A0A8S0PMV2** | **submitted name: Uncharacterized protein [OS=Olea europaea subsp. europaea]** | **2** | **504** | **STTAVLDLLPR** | **-** |
| A0A8S0TWA0 | Filament-like plant protein [OS=Olea europaea subsp. europaea] | 1 | 659 | LQLESIK | - |
| A0A8S0S8S6 | DNA-directed RNA polymerase [OS=Olea europaea subsp. europaea] | 1 | 1038 | IAAEQELIR | Nucleic acid binding activity; DNA-directed 5´-3´RNA polymerase activity |
| A0A8S0SET3 | DUF4005 domain-containing protein [OS=Olea europaea subsp. europaea] | 4 | 188 | RHMVVGER | - |
| **A0A8S0UEF5** | **Structural maintenance of chromosomes protein [OS=Olea europaea subsp. europaea]** | **1** | **1247** | **NEAEAYMLK** | **ATP binding; ATP hydrolysis activity** |
| A0A8S0RLA4 | submitted name: Beta-glucuronosyltransferase 14A-like [OS=Olea europaea subsp. europaea] | 3 | 435 | KFHKDDPVLDK | Glucuronosyltransferase activity |
| A0A8S0TQ48 | submitted name: Probable fructokinase-7 [OS=Olea europaea subsp. europaea] | 3 | 347 | ESELDIDLIK | Kinase activity |
| A0A8S0R5S2 | submitted name: BRANCHLESS TRICHOME [OS=Olea europaea subsp. europaea] | 3 | 322 | LLELENSKR | - |
| A0A8S0RUX3 | submitted name: PREDICTED: uncharacterized protein LOC105974453 [OS=Olea europaea subsp. europaea] | 4 | 178 | YIELNKK | - |
| A0A8S0QK29 | submitted name: 14-3-3 A [OS=Olea europaea subsp. europaea] | 2 | 327 | NLLSVAYK | - |
| A0A8S0RDK1 | submitted name: Zinc finger BED domain-containing RICESLEEPER 2-like [OS=Olea europaea subsp. europaea] | 3 | 211 | ILNLIVK | - |
| A0A8S0TXF9 | submitted name: ADP-ribosylation factor [OS=Olea europaea subsp. europaea] | 4 | 224 | DAVLLVFANK | GTP binding; GTPase activity |
| A0A8S0PHV1 | Arogenate dehydratase [OS=Olea europaea subsp. europaea] | 2 | 420 | LSQSRTAIR | Arogenate dehydratase activity; Prephenate dehydratase activity |
| A0A8S0SNF1 | submitted name: Uncharacterized protein [OS=Olea europaea subsp. europaea] | 6 | 162 | MSTASTLAQR | - |
| A0A8S0R6G9 | Kinesin-like protein [OS=Olea europaea subsp. europaea] | 1 | 657 | IASYIIEQR | Cytoeskeletal activity; ATP binding |
| A0A8S0TKL8 | submitted name: 40S ribosomal S9 [OS=Olea europaea subsp. europaea] | 4 | 195 | LDYVLGLR | Nucleic acid binding activity; Structural constituent of ribosome |
| A0A2K9RSI5 | DNA-directed RNA polymerase subunit beta'' [OS=Olea exasperata] | 1 | 1394 | DPRIPIK | Nucleic acid binding activity; Zinc ion binding |
| A0A8S0VHD2 | submitted name: SRG1-like [OS=Olea europaea subsp. europaea] | 4 | 350 | MADIASATTAPSLDK | 2-oxoglutarate-dependent dioxygenase activity; Metal ion binding |
| A0A8S0UX23 | submitted name: Endo-1,3 1,4-beta-D-glucanase-like [OS=Olea europaea subsp. europaea] | 3 | 241 | SPPELLK | Hydrolase activity |
| A0A8S0QEF9 | submitted name: (S)-ureidoglycine aminohydrolase [OS=Olea europaea subsp. europaea] | 13 | 76 | SGSPPIGVER | Ureidoglycine aminohydrolase activity |
| A0A8S0RWP0 | submitted name: Catalase isozyme 1 [OS=Olea europaea subsp. europaea] | 3 | 296 | LELELGSGR | - |
| A0A8S0Q8A0 | submitted name: Zinc finger ZAT4-like [OS=Olea europaea subsp. europaea] | 3 | 500 | KNLDNESEVNSPKK | - |
| A0A8S0RRT2 | submitted name: IQ-DOMAIN 1 [OS=Olea europaea subsp. europaea] | 3 | 313 | MGSGVWFK | Calmodulin binding |
| A0A8S0PP20 | submitted name: LEAF RUST 10 DISEASE-RESISTANCE LOCUS RECEPTOR-LIKE PROTEIN KINASE-like [OS=Olea europaea subsp. europaea] | 2 | 607 | DITSSNVFVEK | Kinase activity; ATP binding |
| A0A8S0T4M7 | Filament-like plant protein 3 [OS=Olea europaea subsp. europaea] | 2 | 589 | AENETFVLKK | - |
| A0A8S0PFE4 | Dihydrolipoyl dehydrogenase [OS=Olea europaea subsp. europaea] | 2 | 542 | IGVETDKAGR | Dihydrolipoyldehydrogenase activity; Flavin adenine dinucleotide binding |
| A0A8S0QI47 | submitted name: Uncharacterized oxidoreductase [OS=Olea europaea subsp. europaea] | 4 | 359 | DIAVLDAMLESGK | Nucleotide binding |
| A0A8S0UP35 | submitted name: 9-cis-epoxycarotenoid dioxygenase NCED6, chloroplastic [OS=Olea europaea subsp. europaea] | 1 | 570 | SELSEIR | Metal ion binding; Oxidoreductase activity |
| A0A8S0UVB2 | submitted name: ABC transporter B family member 2-like [OS=Olea europaea subsp. europaea] | 1 | 1336 | AGFAGDDAPR; VAPEEHPVLLTEAPLNPK | Transporter activity; ATP binding; ATP hydrolysis activity |
| A0A8S0R0X1 | submitted name: Uncharacterized protein [OS=Olea europaea subsp. europaea] | 6 | 269 | RPTNCESLSSSPDEKFR | - |
| A0A8S0UEA9 | submitted name: Uncharacterized protein [OS=Olea europaea subsp. europaea] | 2 | 612 | LSGQQKKEELER | - |
| A0A8S0UYB9 | Phosphoinositide phospholipase C [OS=Olea europaea subsp. europaea] | 2 | 548 | LSIIGVAADSIK | Phosphatidylinositol phospholipase C activity |
| A0A8S0QI89 | Diphthamide biosynthesis protein 3 [OS=Olea europaea subsp. europaea] | 9 | 80 | IGEEIER | Catalytic activity |
| A0A8S0RR81 | AAA+ ATPase domain-containing protein [OS=Olea europaea subsp. europaea] | 1 | 949 | LSNLNLLGK | ADP binding; ATP hydrolysis activity |
| A0A8S0SB03 | Helicase MAGATAMA 3 [OS=Olea europaea subsp. europaea] | 1 | 1104 | QLPAMVQSEICK | Helicase activity |
| **A0A8S0TEC5** | **submitted name: Heat shock cognate 70 kDa 2-like [OS=Olea europaea subsp. europaea]** | **2** | **679** | **VEIIANDQGNR** | **ATP binding; ATP-dependent protein folding chaperone** |
| A0A8S0PK37 | poly(A)-specific ribonuclease [OS=Olea europaea subsp. europaea] | 3 | 274 | LAELLEVER | Nucleic acid binding activity; Poly(A)-specific ribonuclease activity |

**Table S2.** Amino acid sequences identified in Batch 1. Table includes the following information: Unique identifier assigned to the protein in the UniProtKB database (**Accession**); Description of the protein and its origin (**Description**); Percentage of the protein sequence that aligns with known proteins in the database (**Coverage [%]**; Number of amino acids in the protein sequence (**#Aas);**  Amino acid sequence of the protein (**Sequence**); The function that the protein performs at the molecular level (**Molecular function**). Proteins identified in all three batches are indicated in **bold**.

| **Accession** | **Description** | **Coverage [%]** | **# AAs** | **Sequence** | **Molecular Function** |
| --- | --- | --- | --- | --- | --- |
| **XOS BATCH 1** | | | | | |
| A0A8S0TPK8 | submitted name: Late blight resistance homolog R1A-3 isoform X1 [OS=Olea europaea subsp. europaea] | 6 | 166 | DIEVVANELK | ADP binding |
| **A0A8S0PS52** | **submitted name: Actin-7 [OS=Olea europaea subsp. europaea]** | **7** | **377** | **VAPEEHPVLLTEAPLNPK** | **ATP binding; hydrolase activity** |
| A0A8S0PDC0 | submitted name: Myosin-2 [OS=Olea europaea subsp. europaea] | 7 | 196 | NEENSALKMQLQK | - |
| **A0A8S0SJ95** | **Histone H2A [OS=Olea europaea subsp. europaea]** | **11** | **151** | **HLQLAIR** | **Nucleic acid binding activity; protein heterodimerization activity; structural constituent of chromatin** |
| A0A8S0PBS2 | submitted name: Homeobox-leucine zipper HDG11 [OS=Olea europaea subsp. europaea] | 1 | 719 | LQUENVQLK | Nucleic acid binding activity; RNA polymerase II-Specific; lipid binding |
| A0A8S0S4P8 | MLO-like protein [OS=Olea europaea subsp. europaea] | 1 | 549 | LQAILTR | Calmodulin binding |
| **A0A8S0UR70** | **dolichol kinase [OS=Olea europaea subsp. europaea]** | **2** | **602** | **MAPLLLQLNGER** | **Kinase activity** |
| A0A8S0ST76 | Histone H4 [OS=Olea europaea subsp. europaea] | 5 | 184 | ISGLIYEETR | Nucleic acid binding activity; protein heterodimerization activity; structural constituent of chromatin |
| **A0A8S0R4B0** | **Fructose-bisphosphate aldolase [OS=Olea europaea subsp. europaea]** | **2** | **689** | **GILAADESTGTIGK** | **Fructose-bisphosphate aldolase activity** |
| **A0A8S0SZP0** | **tryptophan synthase [OS=Olea europaea subsp. europaea]** | **3** | **264** | **ESTLHLVLR** | **mRNA binding; structural constituent of ribosome; tyrptophan synthase activity** |
| A0A8S0VNA2 | submitted name: Two-component response regulator ARR18-like [OS=Olea europaea subsp. europaea] | 2 | 505 | KTLGQDIIDTNR | Nucleic acid binding activity |
| **A0A8S0V1P6** | **submitted name: BZIP transcription factor 11-like [OS=Olea europaea subsp. europaea]** | **9** | **135** | **LDDLSAQIAQLR** | **DNA-binding transcription factor activity; protein heterodimerization activity** |
| A0A8S0TDE5 | Sialate O-acetylesterase domain-containing protein [OS=Olea europaea subsp. europaea] | 6 | 205 | RTNVALQDGGQIR | Hydrolase activity |
| A0A8S0ULV8 | submitted name: Glycosyltransferase family 2 [OS=Olea europaea subsp. europaea] | 2 | 930 | DAGSFVLPVDPKDK | - |
| **A0A8S0QCC2** | **submitted name: GTPase Era [OS=Olea europaea subsp. europaea]** | **2** | **437** | **KLEWYEK** | **Nucleic acid binding activity; GTP binding** |
| **A0A8S0PCB3** | **Elongation factor 1-alpha [OS=Olea europaea subsp. europaea]** | **2** | **483** | **IGGIGTVPVGR** | **Nucleic acid binging activity; GTPase activity; Translation elongation factor activity** |
| A0A8S0PAR6 | Elongation factor Tu [OS=Olea europaea subsp. europaea] | 2 | 452 | TTLTAAITR | Nucleic acid binging activity; GTPase activity; Translation elongation factor activity |
| **A0A8S0PMV2** | **submitted name: Uncharacterized protein [OS=Olea europaea subsp. europaea]** | **2** | **504** | **STTAVLDLLPR** | **-** |
| **A0A8S0TEC5** | **submitted name: Heat shock cognate 70 kDa 2-like [OS=Olea europaea subsp. europaea]** | **2** | **679** | **IINEPTAAAIAYGLDK** | **ATP binding; ATP-dependent protein folding chaperone** |
| A0A8S0RR81 | AAA+ ATPase domain-containing protein [OS=Olea europaea subsp. europaea] | 1 | 949 | LSNLNLLGK | ADP binding; ATP hydrolysis activity |
| **A0A8S0QIX0** | **submitted name: Transcription factor bHLH18-like [OS=Olea europaea subsp. europaea]** | **3** | **348** | **FSDKDVLIR** | **Protein dimerization activity** |
| A0A8S0TXF9 | submitted name: ADP-ribosylation factor [OS=Olea europaea subsp. europaea] | 4 | 224 | DAVLLFANK | GTP binding; GTPase activity |
| **A0A8S0QBD8** | **Proteasome subunit alpha type [OS=Olea europaea subsp. europaea]** | **6** | **249** | **LTVEDPVTVEYITR** | **-** |
| **A0A8S0UEF5** | **Structural maintenance of chromosomes protein [OS=Olea europaea subsp. europaea]** | **1** | **1247** | **NEAEAYMLK** | **ATP binding; ATP hydrolysis activity** |
| A0A8S0PN57 | submitted name: Probable mitochondrial adenine nucleotide transporter BTL3 [OS=Olea europaea subsp. europaea] | 2 | 431 | NIFELIK | - |
| A0A8S0PQL8 | submitted name: U3 small nucleolar RNA-associated 25 [OS=Olea europaea subsp. europaea] | 1 | 758 | ADVENIGR | Nucleic acid binding activity |
| A0A8S0UYB9 | Phosphoinositide phospholipase C [OS=Olea europaea subsp. europaea] | 2 | 548 | LSIIGVAADSIK | Phosphatidylinositol phospholipase C activity |
| A0A8S0V262 | submitted name: Uncharacterized protein [OS=Olea europaea subsp. europaea] | 6 | 151 | VNEAQSR | - |
| A0A8S0UA72 | submitted name: Uncharacterized protein [OS=Olea europaea subsp. europaea] | 12 | 108 | AVQISVSPNTIAK | - |
| A0A8S0R6G9 | Kinesin-like protein [OS=Olea europaea subsp. europaea] | 1 | 657 | IASYIIEQR | Cytoeskeletal activity; ATP binidng |
| **A0A8S0SJ95** | submitted name: Actin [OS=Olea europaea subsp. europaea] | 5 | 377 | AGFAGDDAPR; GYSFTTTAER | ATP binding; hydrolase activity |
| A0A8S0SI61 | Starch synthase, chloroplastic/amyloplastic [OS=Olea europaea subsp. europaea] | 2 | 611 | EALQAAVGLPVDK | Glycogen (starch) synthase activity |
| A0A8S0RTD0 | Casein kinase II subunit beta [OS=Olea europaea subsp. europaea] | 5 | 287 | NKLPSSSIPSTSAR | Enzyme regulator activity; kinase activity |
| A0A8S0TDH1 | BSD domain-containing protein [OS=Olea europaea subsp. europaea] | 2 | 422 | TDLAEIGGR | - |
| A0A8S0PVK9 | submitted name: Galactan 1,3-beta-galactosidase [OS=Olea europaea subsp. europaea] | 2 | 474 | LNVLERPK | Hydrolase activity; hydrolyzing O-glucosyl compounds |
| A0A8S0VE99 | submitted name: Phosphatidylinositol phosphatidylcholine transfer SFH8-like isoform X1 [OS=Olea europaea subsp. europaea] | 1 | 697 | LIEGEYER | - |
| A0A8S0RRT2 | submitted name: IQ-DOMAIN 1 [OS=Olea europaea subsp. europaea] | 3 | 313 | MGSGVWFK | Calmodulin binding |
| A0A8S0UEE7 | Starch synthase catalytic domain-containing protein [OS=Olea europaea subsp. europaea] | 2 | 530 | TGGLGDVAGALPK | Glycosyltransferase activity |
| A0A8S0VMP2 | submitted name: Uncharacterized protein [OS=Olea europaea subsp. europaea] | 12 | 86 | MLQDEYALLK | - |

**Table S3.** Amino acid sequences identified in Batch 3. Table includes the following information. Unique identifier assigned to the protein in the UniProtKB database (**Accession**); Description of the protein and its origin (**Description**); Percentage of the protein sequence that aligns with known proteins in the database (**Coverage [%]**; Number of amino acids in the protein sequence (**#Aas);**  Amino acid sequence of the protein (**Sequence**); The function that the protein performs at the molecular level (**Molecular function**). Proteins identified in all three batches are indicated in **bold**.

| **Accession** | **Description** | **Coverage [%]** | **# AAs** | **Sequence** | **Molecular Function** |
| --- | --- | --- | --- | --- | --- |
| **XOS BATCH 3** | | | | | |
| ***A0A8S0PS52** | **submitted name: Actin-7 [OS=Olea europaea subsp. europaea]** | **7** | **377** | **AGFAGDDAPR; VAPEEHPVLLTEAPLNPK** | **ATP binding; Hydrolase activity** |
| **A0A8S0SJ95** | **submitted name: Actin [OS=Olea europaea subsp. europaea]** | **5** | **377** | **AGFAGDDAPR; GYSFTTTAER** | **ATP binding; Hydrolase activity** |
| **A0A8S0SJ95** | **Histone H2A [OS=Olea europaea subsp. europaea]** | **11** | **151** | **HLQLAIR; AGLQFPVGR** | **Nucleic acid binding activity; Protein heterodimerization activity; Structural constituent of chromatin** |
| **A0A8S0SZP0** | **tryptophan synthase [OS=Olea europaea subsp. europaea]** | **3** | **264** | **ESTLHLVLR** | **mRNA binding; Structural constituent of ribosome; Tryptophan synthase activity** |
| **A0A8S0TEC5** | **submitted name: Heat shock cognate 70 kDa 2-like [OS=Olea europaea subsp. europaea]** | **2** | **679** | **IINEPTAAAIAYGLDK** | **ATP binding; ATP-dependent protein folding chaperone** |
| **A0A8S0V1P6** | **submitted name: BZIP transcription factor 11-like [OS=Olea europaea subsp. europaea]** | **9** | **135** | **LDDLSAQIAQLR** | **DNA-binding transcription factor activity; Protein heterodimerization activity** |
| **A0A8S0ST76** | **Histone H4 [OS=Olea europaea subsp. europaea]** | **5** | **184** | **ISGLIYEETR** | **Nucleic acid binding activity; Protein heterodimerization activity; Structural constituent of chromatin** |
| **A0A8S0R4B0** | **Fructose-bisphosphate aldolase [OS=Olea europaea subsp. europaea]** | **2** | **689** | **GILAADESTGTIGK** | **Fructose-bisphosphate aldolase activity** |
| **A0A8S0UR70** | **dolichol kinase [OS=Olea europaea subsp. europaea]** | **2** | **602** | **MAPLLLQLNGER** | **Kinase activity** |
| **A0A8S0QBD8** | **Proteasome subunit alpha type [OS=Olea europaea subsp. europaea]** | **6** | **249** | **LTVEDPVTVEYITR** | **-** |
| A0A8S0PAR6 | Elongation factor Tu [OS=Olea europaea subsp. europaea] | 2 | 452 | TTLTAAITR | Nucleic acid binding activity; GTPase activity; Translation elongation factor activity |
| A0A8S0RZ67 | submitted name: Uncharacterized protein [OS=Olea europaea subsp. europaea] | 6 | 250 | MGDLAANLLLVSDLR | - |
| A0A8S0TDE5 | Sialate O-acetylesterase domain-containing protein [OS=Olea europaea subsp. europaea] | 6 | 205 | RTNVALQDGGQIR | Hydrolase activity |
| A0A8S0R5A7 | FLZ-type domain-containing protein [OS=Olea europaea subsp. europaea] | 7 | 167 | MRNLSVSPNLR | - |
| ***A0A8S0UEF5** | **Structural maintenance of chromosomes protein [OS=Olea europaea subsp. europaea]** | **1** | **1247** | **NEAEAYMLK** | **ATP binding; ATP hydrolysis activity** |
| **A0A8S0QIX0** | **submitted name: Transcription factor bHLH18-like [OS=Olea europaea subsp. europaea]** | **3** | **348** | **FSDKDVLIR** | **Protein dimerization activity** |
| A0A8S0SMR3 | submitted name: SCAI [OS=Olea europaea subsp. europaea] | 1 | 749 | STELLIR | Nucleic acid binding activity; Protein heterodimerization activity; Structural constituent of chromatin; Transcription corepressor activity |
| **A0A8S0PCB3** | **Elongation factor 1-alpha [OS=Olea europaea subsp. europaea]** | **2** | **483** | **IGGIGTVPVGR** | **Nucleic acid binding activity; GTPase activity; Translation elongation factor activity** |
| **A0A8S0QCC2** | **submitted name: GTPase Era [OS=Olea europaea subsp. europaea]** | **2** | **437** | **KLEWYEK** | **Nucleic acid binding activity** |
| A0A8S0RDK1 | submitted name: Zinc finger BED domain-containing RICESLEEPER 2-like [OS=Olea europaea subsp. europaea] | 3 | 211 | ILNLIVK | - |
| ***A0A8S0PMV2** | **submitted name: Uncharacterized protein [OS=Olea europaea subsp. europaea]** | **2** | **504** | **STTAVLDLLPR** | **-** |
| A0A8S0SIQ0 | submitted name: Nuclease HARBI1 [OS=Olea europaea subsp. europaea] | 2 | 390 | EITEIQNK | Nucleic acid binding activity; Metal ion binding; Nuclease activity |
| A0A8S0TPK8 | submitted name: Late blight resistance homolog R1A-3 isoform X1 [OS=Olea europaea subsp. europaea] | 6 | 166 | DIEVVANELK | ADP binding |
| A0A8S0SHT5 | submitted name: Probable transcription factor 21 [OS=Olea europaea subsp. europaea] | 4 | 201 | LELQTLER | DNA-binding transcription factor activity |
| A0A8S0PFS0 | Receptor-like serine/threonine-protein kinase [OS=Olea europaea subsp. europaea] | 1 | 801 | RVQIICGIAR | Kinase activity; ATP binding |
| A0A8S0VMP2 | submitted name: Uncharacterized protein [OS=Olea europaea subsp. europaea] | 12 | 86 | MLQDEYALLK | - |
| A0A8S0QI29 | submitted name: Tetratricopeptide repeat 7A-like [OS=Olea europaea subsp. europaea] | 1 | 731 | LEGGFNLK | - |
| A0A8S0QI47 | submitted name: Uncharacterized oxidoreductase [OS=Olea europaea subsp. europaea] | 4 | 359 | DIAVLDAMLESGK | Nucleotide binding |
| A0A8S0TH01 | submitted name: Disease resistance At4g27190-like [OS=Olea europaea subsp. europaea] | 5 | 295 | TVEHIPGPSIMSQK | ADP binding |
| ***A0A8S0PS52** | submitted name: Actin-7 [OS=Olea europaea subsp. europaea] | 7 | 377 | AGFAGDDAPR; VAPEEHPVLLTEAPLNPK | ATP binding; Hydrolase activity |
| ***A0A8S0UEF5** | **Structural maintenance of chromosomes protein [OS=Olea europaea subsp. europaea]** | **1** | **1247** | **NEAEAYMLK** | **ATP binding; ATP hydrolysis activity** |
| A0A8S0RWP0 | submitted name: Catalase isozyme 1 [OS=Olea europaea subsp. europaea] | 3 | 296 | LELELGSGR | - |
| ***A0A8S0PMV2** | **submitted name: Uncharacterized protein [OS=Olea europaea subsp. europaea]** | **2** | **504** | **STTAVLDLLPR** | **-** |

*Accessions marked with an asterisk appeared more than once in the dataset and were counted as single entries for protein quantification
